# Aerobic exercise preconditioning inhibits GSDME-dependent pyroptosis in myocardial ischemia–reperfusion injury through IGFBP2-AKT-PDCD4 signaling

**DOI:** 10.1186/s43556-026-00633-y

**Published:** 2026-11-05

**Authors:** Yi Li, Xiang Wang, Yejing Zhao, Yunyi Gao, Chenxi Xia, Jun Wang, Jiefu Yang, Chenguang Yang, Fang Wang

**Affiliations:** 1 Department of Cardiology, Beijing Hospital, National Center of Gerontology, Institute of Geriatric Medicine, Chinese Academy of Medical Sciences, Beijing, 100730 People’s Republic of China; 2 Chinese Academy of Medical Sciences and Peking Union Medical College, Beijing, 100730 People’s Republic of China; 3 Department of Cardiology, Beijing Anzhen Hospital, Capital Medical University, Beijing, 100029 China; 4 Safe Transfusion Lab, Beijing Red Cross Blood Center, Beijing, 100088 China

**Keywords:** Ischemia–reperfusion, GSDME, IGFBP2, Pyroptosis, Aerobic exercise, Mitochondium

## Abstract

Restoring blood flow to ischemic myocardium is essential for tissue survival but can itself cause additional injury. Mitochondrial dysfunction and pyroptosis, an inflammatory form of cell death, contribute to this reperfusion injury. Although aerobic exercise protects the heart, how exercise preconditioning engages circulating signals to preserve mitochondria and limit pyroptosis remains unclear. Here we show that insulin-like growth factor-binding protein 2 (IGFBP2) contributes to exercise-induced cardioprotection through IGF-1R/PI3K/AKT signaling and suppression of programmed cell death protein 4 (PDCD4). Human plasma proteomics identified elevated IGFBP2 in individuals with sustained aerobic exercise habits, and experiments in mice and primary cardiomyocytes linked this pathway to mitochondrial preservation and reduced gasdermin E (GSDME)-dependent pyroptosis. A 12-week exercise programme before ischemia–reperfusion improved cardiac function and reduced infarct size, whereas global *Igfbp2* knockout or cardiomyocyte-specific *Igf1r* deficiency largely abolished these benefits. Genetic and pharmacological perturbations supported PDCD4 suppression as a downstream mechanism of IGFBP2 signaling. GSDME deletion reduced cell injury without preventing PDCD4-associated mitochondrial dysfunction, supporting a model in which mitochondrial damage acts upstream of GSDME activation. These findings connect an exercise-responsive circulating factor to mitochondrial protection and downstream control of inflammatory cell death, advancing mechanistic understanding of exercise-induced cardiac resilience. They identify IGFBP2 as an important mediator of this protection, while other exercise-responsive pathways may also contribute. This pathway provides a rationale for investigating approaches to limit reperfusion injury, although its therapeutic relevance requires further validation.

**Supplementary Information:**

The online version contains supplementary material available at https://doi.org/10.1186/s43556-026-00633-y.

## Introduction

In a contemporary Chinese cohort of patients hospitalized with ST-segment elevation myocardial infarction, de novo heart failure occurred in 10.6% of patients and was associated with increased short-term mortality [1]. Myocardial ischemia–reperfusion (I/R) injury refers to the paradoxical tissue damage that occurs when blood flow is restored after a period of coronary occlusion. Although reperfusion is essential to salvage ischemic myocardium, the sudden reintroduction of oxygen and substrates triggers excessive reactive oxygen species generation, calcium overload, mitochondrial dysfunction, and inflammatory responses. These events promote cardiomyocyte loss through various mechanisms. In recent years, various forms of programmed cell death (PCD) have been recognized as key mechanisms underlying the pathophysiology of myocardial I/R injury [2–5]. Among these, pyroptosis—a form of inflammatory programmed death—has gained increasing attention due to its potent pro-inflammatory effects and role in exacerbating myocardial injury [6–9]. In our previous studies, we demonstrated that Gasdermin E (GSDME)-dependent pyroptosis is an important mechanism for myocardial injury induced by I/R [10]. GSDME-dependent pyroptosis is characterized by cleavage of GSDME [7, 10, 11]. Therefore, targeting GSDME-dependentpyroptotic pathways may serve as a therapeutic approach for alleviating cardiomyocyte I/R injury.

Aerobic exercise is well established as an effective, non-pharmacological intervention to improve cardiovascular health and reduce the incidence and severity of cardiovascular events [12–14]. In patients with recent myocardial infarction and reduced left ventricular ejection fraction, participation in structured cardiac rehabilitation was associated with improved post-exercise systolic blood pressure and heart rate recovery [15]. Ample evidence supports the notion that regular aerobic exercise enhances myocardial resistance to ischemic insults and mitigates the extent of I/R injury [16–19]. In our previous Molecular Medicine study, aerobic exercise began 1 week after I/R and continued for 8 weeks; this post-I/R exercise regimen attenuated GSDME-dependent pyroptosis and promoted cardiac recovery [10]. The present study investigates exercise preconditioning, defined here as 12 weeks of exercise before I/R, with a focus on how circulating IGFBP2 engages PI3K/AKT-PDCD4 signaling to preserve mitochondria and limit GSDME-dependent pyroptosis.

Insulin-like Growth Factor Binding Protein 2 (IGFBP2) is a multifunctional protein involved in the regulation of cell proliferation, differentiation, and apoptosis [7], and has been linked to various disease states, including cardiovascular pathology [10, 20]. Our previous studies suggested that during recovery phase, IGFBP2 played a protective role in myocardial I/R injury, potentially through modulation of pyroptosis and ferroptosis [10, 21]. Programmed Cell Death Protein 4 (PDCD4) is a pro-death factor that has been associated with mitochondrial injury and stress signaling [22, 23]. These observations raise the possibility that PDCD4 may participate in the regulation of GSDME activation and pyroptotic cell death in the heart.

Building on this background, the present study aimed to elucidate the mechanisms by which aerobic exercise preconditioning mitigates myocardial I/R injury. Specifically, we hypothesize that aerobic exercise-induced upregulation of circulating IGFBP2 activates the IGF-1R/PI3K/AKT pathway, leading to downregulation of *Pdcd4* expression and subsequent inhibition of *Gsdme*-mediated pyroptosis. To test this hypothesis, we integrated human plasma and mouse myocardial proteomics with genetic and pharmacological perturbations in complementary in vivo and in vitro I/R models.

## Results

### Long-term aerobic exercise preconditioning improves myocardial I/R injury and attenuates GSDME-dependent pyroptosis

**Fig. 1:**
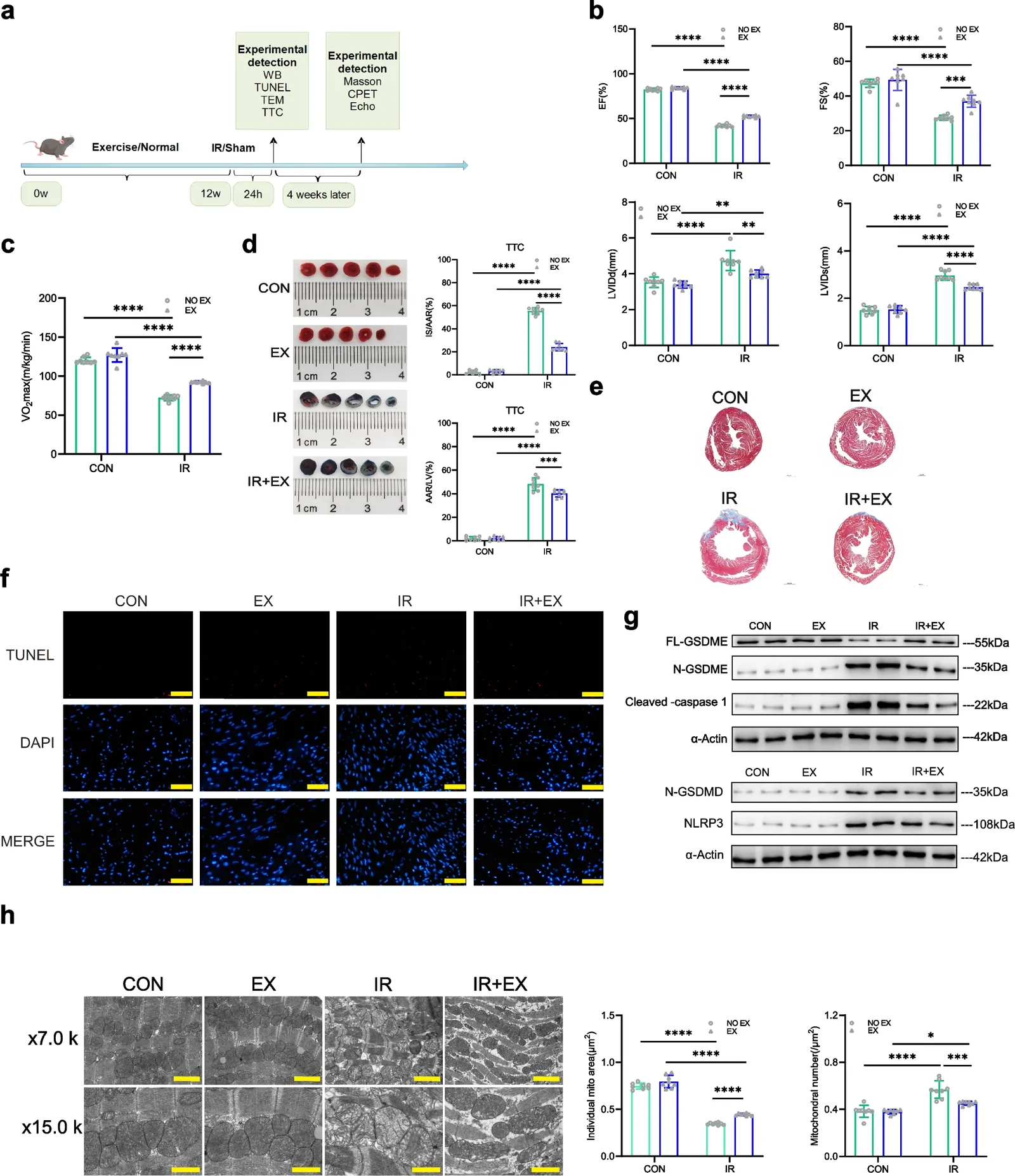
Long-term aerobic exercise preconditioning attenuates myocardial ischemia–reperfusion injury and GSDME-associated pyroptotic signaling. **a** Schematic of the in vivo study design. Mice underwent 12 weeks of aerobic exercise preconditioning before sham surgery or myocardial ischemia–reperfusion (I/R), followed by the indicated tissue collection and functional, histological, molecular, and ultrastructural analyses. **b** Echocardiographic measurements of ejection fraction (EF), fractional shortening (FS), left ventricular internal diameter at end-diastole (LVIDd), and left ventricular internal diameter at end-systole (LVIDs) in control (CON) and I/R mice with or without exercise (EX). **c** Maximal oxygen consumption (VO₂max) in the indicated groups. **d** Representative 2,3,5-triphenyltetrazolium chloride (TTC)-stained heart sections collected 24 h after I/R and quantification of infarct size relative to the area at risk (IS/AAR). **e** Representative Masson's trichrome staining of myocardial fibrosis 4 weeks after I/R. **f** Representative terminal deoxynucleotidyl transferase dUTP nick-end labeling (TUNEL) staining of myocardial sections. Scale bar, 50 µm. **g** Immunoblot analysis of full-length GSDME (FL-GSDME), N-terminal GSDME (N-GSDME), cleaved caspase-1, N-GSDMD, and NLRP3 in myocardial tissue. Corresponding densitometric quantification and statistical comparisons for N-GSDME, cleaved caspase-1, N-GSDMD, and NLRP3 are provided in Fig. S1c. **h** Representative transmission electron microscopy (TEM) images and quantification of individual mitochondrial area and mitochondrial number per unit area. Scale bars, 2 µm (7.0 K) and 1 µm (15.0 K). For quantitative panels, data are presented as mean ± SD (*n* = 8 per group). Statistical significance was determined by two-way ANOVA followed by Tukey's multiple-comparison test. *P < 0.05, **P < 0.01, ***P < 0.001, and ****P < 0.0001; ns, not significant

Our previous study showed that exercise initiated after I/R promoted cardiac recovery by attenuating GSDME-dependent pyroptosis [10]. To evaluate protection induced before ischemic injury, C57BL/6 J mice underwent 12 weeks of aerobic exercise preconditioning or remained sedentary before sham surgery or myocardial I/R (Fig. 1a). Exercise preconditioning improved cardiac function and cardiopulmonary functional capacity (Fig. 1b-c), reduced the IS/AAR ratio and myocardial fibrosis (Fig. 1d-e; Fig. S1a), and reduced TUNEL-positive cells (Fig. 1f; Fig. S1b). Immunoblotting and the corresponding densitometric analyses showed reduced N-GSDME after exercise preconditioning (Fig. 1g; Fig. S1c). Quantified cleaved caspase-1, N-GSDMD, and NLRP3 levels were also lower in the exercise-preconditioned I/R group (Fig. S1c). Exercise also reduced IL-1β and IL-18 levels (Fig. S1d-e) and preserved mitochondrial ultrastructure (Fig. 1h). These findings support attenuation of GSDME-associated pyroptotic signaling but do not establish an exclusive effect on the GSDME pathway. Subsequent mechanistic experiments focused on the contribution of GSDME.

### Long-term aerobic exercise increases circulating IGFBP2 levels

**Fig. 2:**
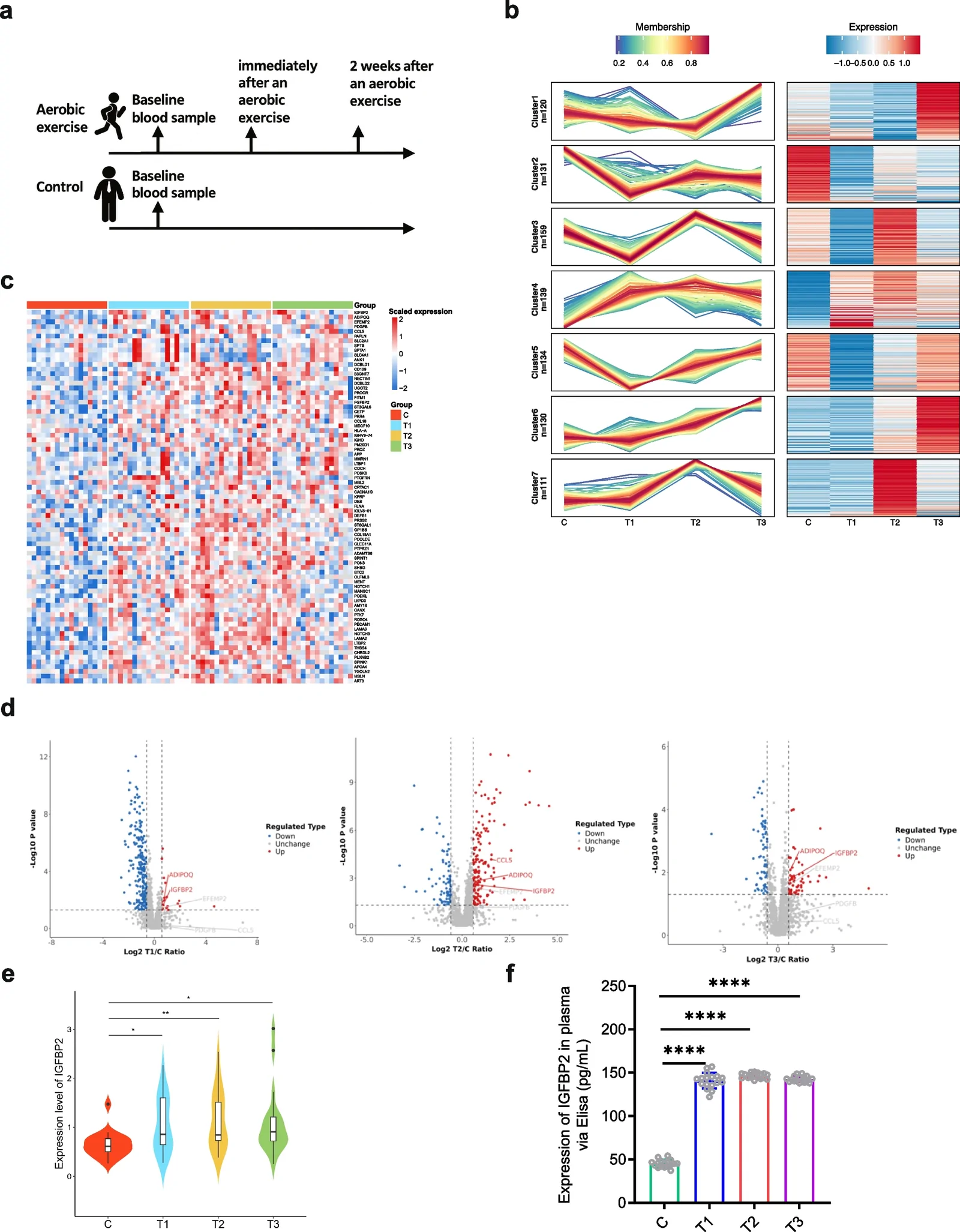
Long-term aerobic exercise is associated with increased plasma IGFBP2 in healthy adults. **a** Study design and serial plasma collection at baseline, immediately after aerobic exercise, and 2 weeks after aerobic exercise. **b** Temporal expression-pattern clustering and corresponding heatmaps across C, T1, T2, and T3. **c** Heatmap of features in the indicated temporal-expression cluster. **d** Volcano plots of differentially expressed features at T1, T2, and T3 relative to C. **e** Violin plots of IGFBP2 abundance across the four plasma-sample groups (*n* = 17). **f** Enzyme-linked immunosorbent assay (ELISA) measurements of plasma IGFBP2 concentrations in 17 independent control participants and at T1, T2, and T3 in the same 17 exercise participants. Data are presented as mean ± SD. C, control-group baseline; T1, aerobic-exercise-group baseline; T2, immediately after aerobic exercise; T3, 2 weeks after aerobic exercise. In panel e–f, T1-T3 were analyzed using one-way repeated-measures ANOVA with the Geisser-Greenhouse correction, followed by Tukey-adjusted pairwise comparisons using pair-specific error estimates without assuming sphericity. Comparisons between the independent control group and each exercise time point used two-sided Welch’s t-tests with Holm adjustment across the three comparisons. The overall time effect was significant (*F*(1.447, 23.150) = 3.799, *P* = 0.0498), whereas all Tukey-adjusted pairwise comparisons were nonsignificant (T1-T2, *P* = 0.0954; T1-T3, *P* = 0.5189; T2-T3, *P* = 0.0689). All three comparisons with the control group had Holm-adjusted *P* < 0.0001. *P < 0.05, **P < 0.01, ***P < 0.001, and ****P < 0.0001; ns, not significant

To identify candidate exerkines that mediate the cardioprotective effects of aerobic exercise preconditioning, we performed untargeted plasma proteomics in healthy individuals with or without sustained aerobic exercise (Fig. 2a). Age and BMI did not differ significantly between groups, whereas VO₂max and anaerobic threshold were higher in the exercise group (Table S1). Plasma proteins were grouped by 7 clusters according to their trend of change pre- or post- one-time exercise. Cluster 4 showed an overall pattern of higher protein abundance in individuals with habitual aerobic exercise than in controls. IGFBP2 was one of the most upregulated proteins in cluster 4 (Fig. 2b-e), and its cardio protection effect was verified in post-I/R phase by our previous studies [10, 21]. ELISA measurements confirmed higher plasma IGFBP2 concentrations at T1, T2, and T3 than in the independent control group (all Holm-adjusted *P* < 0.0001). Within the exercise group, repeated-measures analysis identified an overall time effect (*F*(1.447, 23.150) = 3.799, *P* = 0.0498). However, none of the pairwise differences between T1, T2, and T3 reached statistical significance after Tukey adjustment (T1-T2, *P* = 0.0954; T1-T3, *P* = 0.5189; T2-T3, *P* = 0.0689; Fig. 2f). Together, these findings identify circulating IGFBP2 as an exercise-responsive candidate that may contribute to exercise-induced cardioprotection.

### IGFBP2 contributes to the cardioprotective effects of aerobic exercise preconditioning

**Fig. 3:**
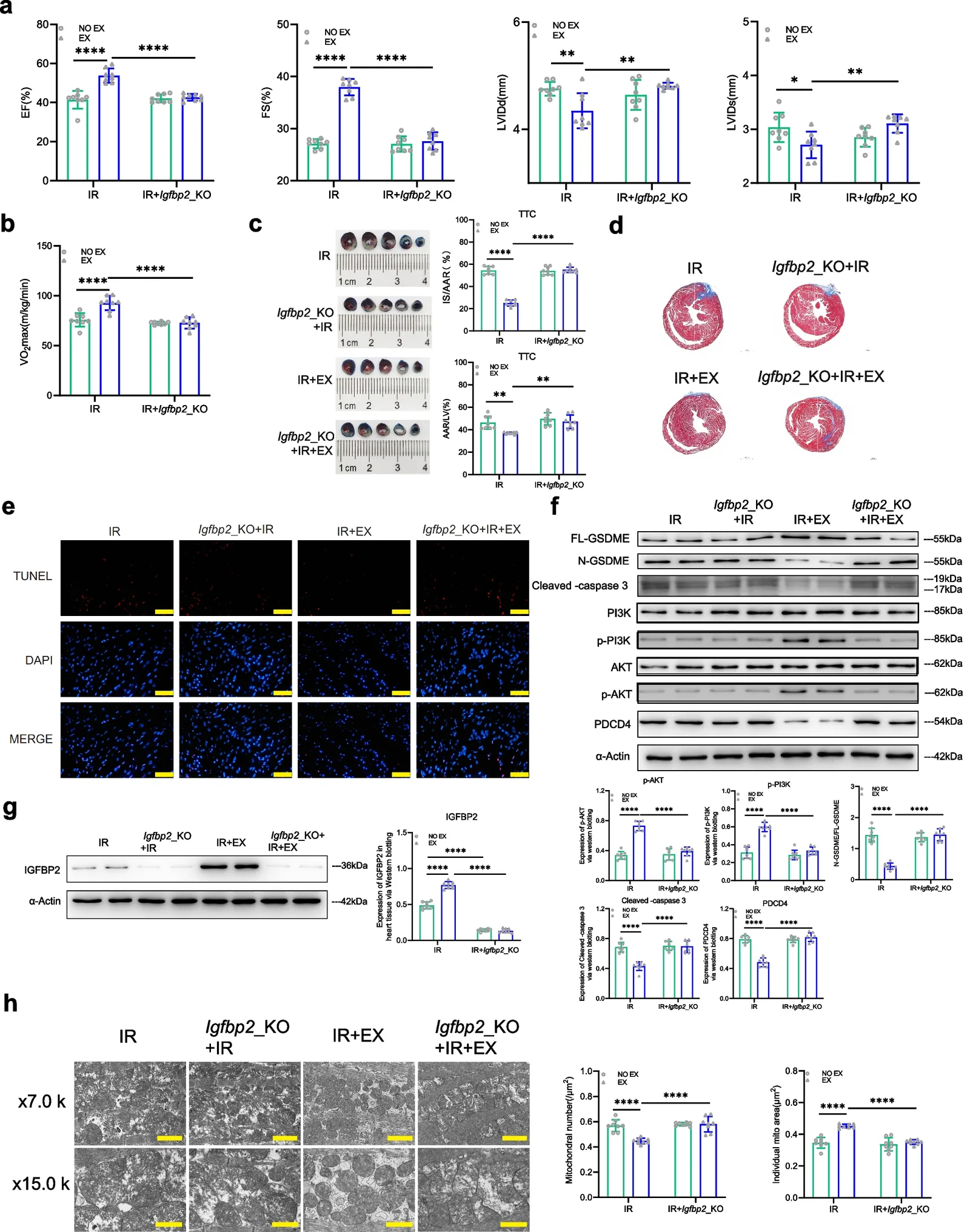
IGFBP2 is required for exercise-mediated cardioprotection against myocardial ischemia–reperfusion injury. **a** Echocardiographic measurements of EF, FS, LVIDd, and LVIDs in I/R, *Igfbp2*-KO + I/R, I/R + EX, and *Igfbp2*-KO + I/R + EX mice. **b** VO₂max in the indicated groups. **c** Representative TTC-stained heart sections collected 24 h after I/R and quantification of infarct size relative to the area at risk (IS/AAR). **d** Representative Masson's trichrome staining of myocardial fibrosis 4 weeks after I/R. **e** Representative TUNEL staining of myocardial sections. Scale bar, 50 µm. **f** Immunoblot analysis of FL-GSDME, N-GSDME, cleaved caspase-3, total and phosphorylated PI3K and AKT, and PDCD4 in myocardial tissue. **g** IGFBP2 immunoblotting and densitometric quantification of IGFBP2 and the indicated signaling proteins. **h** Representative TEM images and quantification of mitochondrial number and individual mitochondrial area. Scale bars, 2 µm (7.0 K) and 1 µm (15.0 K). For quantitative panels, data are presented as mean ± SD (*n* = 8 per group). Statistical significance was determined by two-way ANOVA followed by Tukey's multiple-comparison test. *P < 0.05, **P < 0.01, ***P < 0.001, and ****P < 0.0001; ns, not significant. KO, knockout

To examine the contribution of IGFBP2 to cardioprotection induced by aerobic exercise preconditioning, we subjected full genetic *Igfbp2*-knockout (*Igfbp2*-KO) and wild-type (WT) mice to the myocardial I/R model with or without the aerobic exercise preconditioning protocol described above. Compared with the EX + I/R group, *Igfbp2-*KO abrogated the protective benefits of aerobic exercise preconditioning, manifested by deteriorated cardiac function (Fig. 3a), reduced VO₂max (Fig. 3b), higher IS/AAR ratio (Fig. 3c), aggravated myocardial fibrosis (Fig. 3d; Fig. S2a), increased GSDME-dependent pyroptosis (Fig. 3e-f; Fig. S2b-d) and immunoblotting further verified markedly reduced IGFBP2 levels in both plasma and myocardial tissue in *Igfbp2*-KO mice, regardless of exercise preconditioning (Fig. 3g). Transmission electron microscopy revealed more severe mitochondrial ultrastructural disruption in the *Igfbp2*-KO group (Fig. 3h). Together, these results support an important mediating role for IGFBP2 in exercise-induced protection against myocardial I/R injury in this mouse model.

In vitro, primary cardiomyocytes were subjected to H/R at the cellular level, whereas IGFBP2 pretreatment improved viability and attenuated GSDME-dependent pyroptosis (Fig. S2e-l). Consistently, H/R markedly increased membrane N-GSDME level, whereas IGFBP2 pretreatment reduced this trend (Fig. S3a-b). PI staining further showed that H/R substantially increased the rupture of membrane, which was significantly attenuated by IGFBP2 pretreatment (Fig. S3c). IGFBP2 also preserved mitochondrial integrity and function and inhibited oxidative stress after H/R (Fig. S3d-h). Notably, cardiomyocyte-specific GSDME deletion alleviated H/R-induced GSDME-dependent pyroptosis but did not noticeably improve mitochondrial injury. IGFBP2 continued to protect mitochondria in GSDME-deficient cardiomyocytes, although no further reduction in GSDME-dependent pyroptosis was observed. These findings are consistent with mitochondrial protection acting upstream of GSDME activation, rather than resulting solely from inhibition of GSDME-dependent cell death.

### IGFBP2 mediates protection effect of aerobic exercise preconditioning in I/R myocardium via IGF-1R

On the basis of our previous work implicating IGF-1R in the benefits of aerobic exercise during post-I/R recovery, we next investigated whether IGF-1R is also required for the protection conferred by aerobic exercise preconditioning [10]. To this end, we generated myocardiac-specific *Igf1r* conditional knockout (*Igf1r*-CKO) mice and subjected them to myocardial I/R with or without the aerobic exercise preconditioning; efficient deletion was confirmed by immunoblotting (Fig. S4a). Co-immunoprecipitation showed that IGFBP2 physically interacts with IGF-1R, and this interaction was strengthened by aerobic exercise preconditioning (Fig. S4b). Functionally, both cardiac *Igf-1r* deficiency and *Igfbp2* knockout largely abrogated aerobic exercise preconditioning-induced cardioprotection after I/R injury, as shown by worsened cardiac function, reduced VO₂_max_, increased IS/AAR ratio, aggravated myocardial fibrosis, enhanced TUNEL positivity, and elevated IL-1β and IL-18 levels (Fig. S4c-j; Fig. S5a-b). Consistently, western blotting showed either cardiac *Igf-1r* deficiency or *Igfbp2* knockout increased N-GSDME, cleaved caspase-3, and PDCD4 expression (Fig. S5c-d).

### Aerobic exercise preconditioning activates the PI3K/AKT pathway and suppresses PDCD4

**Fig. 4:**
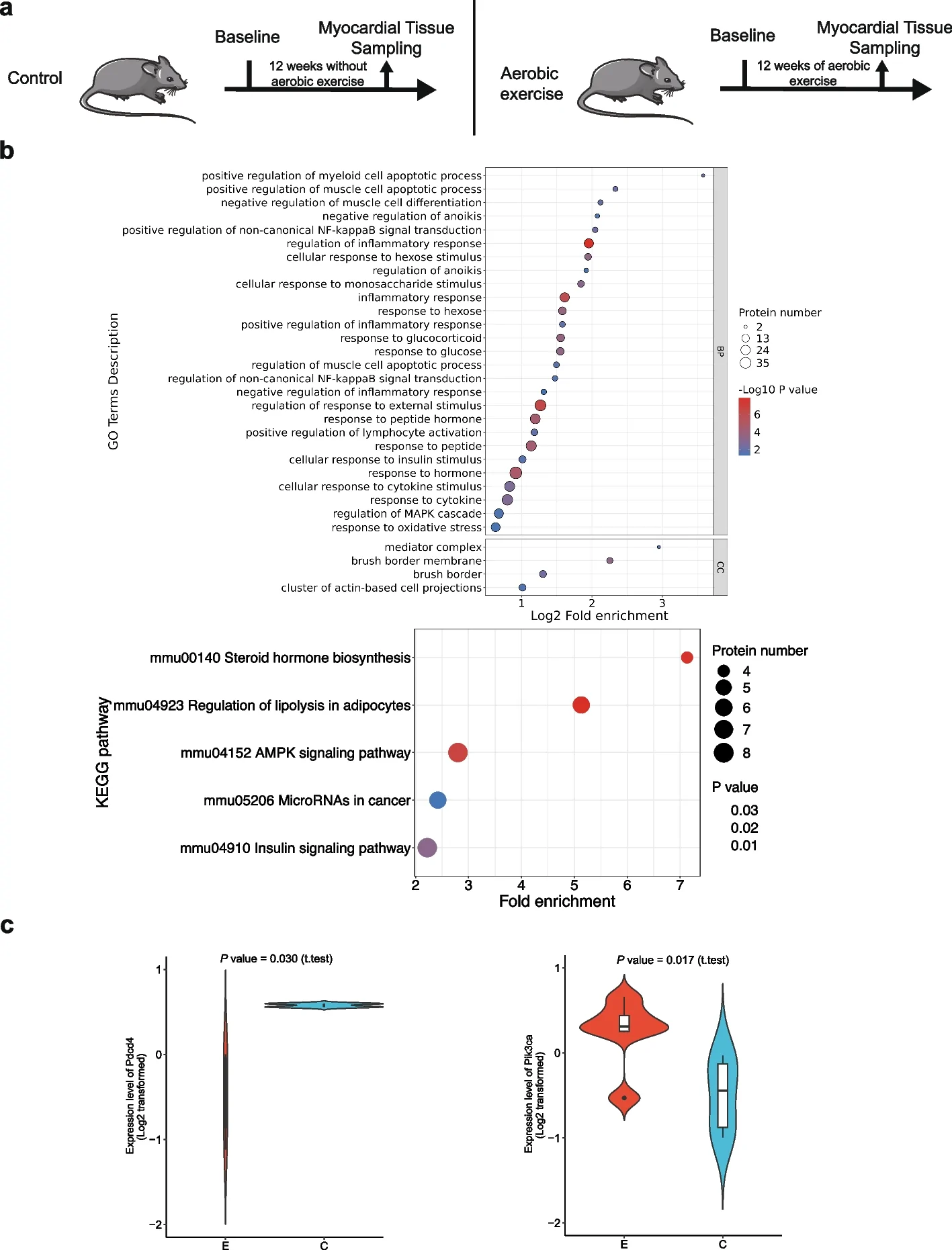
Aerobic exercise alters myocardial PI3K-pathway-associated protein abundance and reduces PDCD4 abundance. **a** Experimental design for 12 weeks of aerobic exercise and myocardial tissue collection for proteomic analysis. **b** Gene Ontology biological-process and cellular-component enrichment analyses and Kyoto Encyclopedia of Genes and Genomes pathway enrichment analysis of proteins altered by aerobic exercise. Bubble size represents the number of proteins, and color represents statistical significance, as indicated. **c** Violin plots of log₂-transformed *Pdcd4* and *Pik3ca* protein abundance in exercise (E) and control (C) myocardial samples (*n* = 8 per group). Statistical significance in panel c was determined by Student's t test; exact *P* values are shown in the figure

To further investigate the intracellular mechanisms by which aerobic exercise preconditioning protects the myocardium from I/R injury, we examined signaling changes associated with aerobic exercise (Fig. 4a). Untargeted proteomic profiling of cardiac tissue from aerobic exercised mice identified reduced *Pdcd4* expression alongside increased *Pik3ca* expression (Fig. 4b-c). Consistently, immunoblotting demonstrated enhanced phosphorylation of PI3K and AKT accompanied by decreased PDCD4 protein levels in the EX + I/R group in vivo (Fig. 3f). Besides, both *Igfbp2* knockout and *Igf-1r* deficiency abrogated the upregulation of PI3K/AKT phosphorylation by exercise (Fig. S5c-d). In primary cardiomyocytes subjected to H/R in vitro, IGFBP2 pretreatment reduced PDCD4 levels and increased PI3K and AKT phosphorylation in both GSDME-sufficient and GSDME-deficient cells (Fig. S2h-i). And *Igf-1r* deficiency abrogated the upregulation of PI3K/AKT phosphorylation by IGFBP2 (Fig. S5e). To examine PDCD4 promoter activity, we performed a reporter assay in HEK-293 T cells, a heterologous cell line, using the PDCD4 promoter-driven firefly luciferase construct pGL3-PDCD4 promoter; PI3K activation reduced reporter activity (Fig. S5f). Collectively, these findings support IGFBP2-dependent activation of IGF-1R/PI3K/AKT signaling and suppression of PDCD4. In vitro, IGFBP2-mediated PDCD4 suppression persisted in GSDME-deficient cardiomyocytes, indicating that this response did not require GSDME under the H/R conditions tested. GSDME independence was not established in the in vivo I/R model.

### IGFBP2 Suppressed PDCD4 by Activating PI3K/AKT to Protect post-I/R Myocardial Mitochondria to Inhibit GSDME-dependent Pyroptosis in I/R Myocardium

To determine whether PI3K/AKT signaling mediates the regulatory effects of IGFBP2 on PDCD4 and GSDME-dependent pyroptosis, primary cardiomyocytes subjected to H/R were pretreated with IGFBP2, together with either a PI3K agonist or antagonist to modulate PI3K activity. PI3K agonist largely simulated the cytoprotective effects of IGFBP2, whereas PI3K antagonist markedly weakened IGFBP2 pretreatment-mediated protection following H/R. Specifically, the PI3K agonist improved post-H/R cell viability to a similar extent as exogenous IGFBP2, while the PI3K antagonist significantly blunted the IGFBP2-induced increase in viability (Fig. S6a). IGFBP2 treatment and PI3K activation reduced TUNEL positivity and the levels of N-GSDME and cleaved caspase-3 and PI3K blockade reversed these changes (Fig. S6b-h). TUNEL was interpreted as a measure of DNA fragmentation, with evidence of GSDME-associated pyroptotic signaling provided by the accompanying GSDME measurements. Besides, IGFBP2 treatment and PI3K activation preserved mitochondrial function after H/R, as evidenced by improved OCR/OXPHOS activity, reduced ROS accumulation, and maintenance of mitochondrial membrane potential on JC-1 staining. In contrast, PI3K inhibition largely reversed the mitochondrial and myocardium protection effects of IGFBP2 (Fig. S7a-e). Mechanistically, IGFBP2 pretreatment and PI3K agonist increased PI3K and AKT phosphorylation and reduced PDCD4. These molecular changes were reversed upon PI3K antagonism (Fig. S6d-e). Collectively, these data indicate that IGFBP2 activates the PI3K/AKT pathway to suppress PDCD4 and preserve mitochondrial function, consistent with reduced downstream GSDME activation and pyroptosis following H/R.

**Fig. 5:**
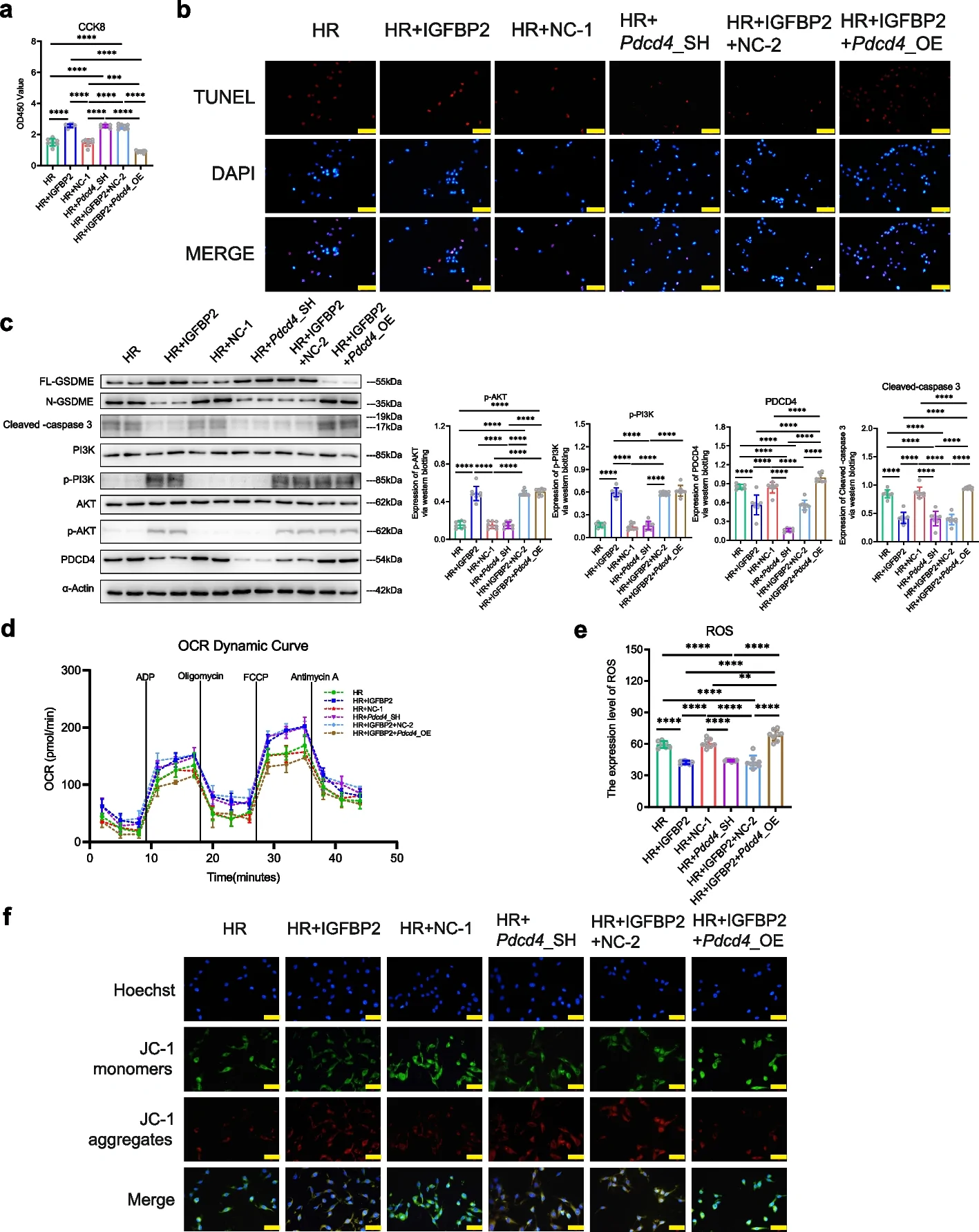
IGFBP2 suppresses PDCD4 to limit GSDME-associated cell death and preserve mitochondrial function in hypoxia-reoxygenation-treated cardiomyocytes. Primary cardiomyocytes were assigned to H/R, H/R + IGFBP2, H/R + NC-1, H/R + *Pdcd4*-SH, H/R + IGFBP2 + NC-2, or H/R + IGFBP2 + *Pdcd4*-OE groups. **a** Cell viability assessed by CCK-8. (b) Representative TUNEL staining. Scale bar, 100 µm. **c** Immunoblot analysis of FL-GSDME, N-GSDME, cleaved caspase-3, total and phosphorylated PI3K and AKT, and PDCD4, with densitometric quantification of the indicated proteins. **d** Oxygen-consumption-rate (OCR) profiles during the mitochondrial stress test. **e** Intracellular reactive oxygen species (ROS) levels. **f** Representative JC-1 fluorescence images showing nuclei (Hoechst), JC-1 monomers (green), JC-1 aggregates (red), and merged images. Scale bar, 50 µm. For quantitative panels, data are presented as mean ± SD (*n* = 8 per group). Statistical significance was determined by one-way ANOVA followed by Tukey's multiple-comparison test. *P < 0.05, **P < 0.01, ***P < 0.001, and ****P < 0.0001; ns, not significant. H/R, hypoxia-reoxygenation; NC, matched negative control; SH, knockdown; OE, overexpression

To investigate whether PDCD4 acts downstream of IGFBP2 in modulating GSDME-dependent pyroptosis, primary cardiomyocytes were pretreated with recombinant IGFBP2 or subjected to *Pdcd4* overexpression or silence modulated by adenovirus (ADV) vectors. CCK-8 results showed that IGFBP2 pretreatment significantly improved cell viability under H/R stress, but this benefit was largely abolished by *Pdcd4* overexpression; conversely, *Pdcd4* silence restored cell viability as of the presence of IGFBP2 (Fig. 5a). TUNEL-positive cells were reduced by IGFBP2 pretreatment, increased by *Pdcd4* overexpression, and reduced by *Pdcd4* silencing (Fig. 5b; Fig. S7f). Immunoblotting showed that IGFBP2 pretreatment reduced cleaved GSDME and cleaved caspase-3 levels and PDCD4 expression, but enhanced PI3K and AKT phosphorylation; these effects were reversed by *Pdcd4* overexpression and restored by *Pdcd4* silencing (Fig. 5c). Mitochondrial assays indicated that IGFBP2 helped maintain mitochondrial function, reduce oxidative stress, and preserve mitochondrial membrane potential; these effects were reversed by *Pdcd4* overexpression and restored by *Pdcd4* silencing (Fig. 5d-f; Fig. S7g-h). In parallel, IGFBP2 pretreatment decreased the release of IL-1β, IL-18, and LDH; these protective effects were abolished by *Pdcd4* overexpression and restored by *Pdcd4* silencing (Fig. S7i-k). Together, these results support a model in which IGFBP2 downregulates PDCD4 and preserves mitochondrial integrity, thereby limiting downstream GSDME activation and pyroptosis.

### GSDME deletion limits PDCD4-associated cell injury without restoring mitochondrial function

**Fig. 6:**
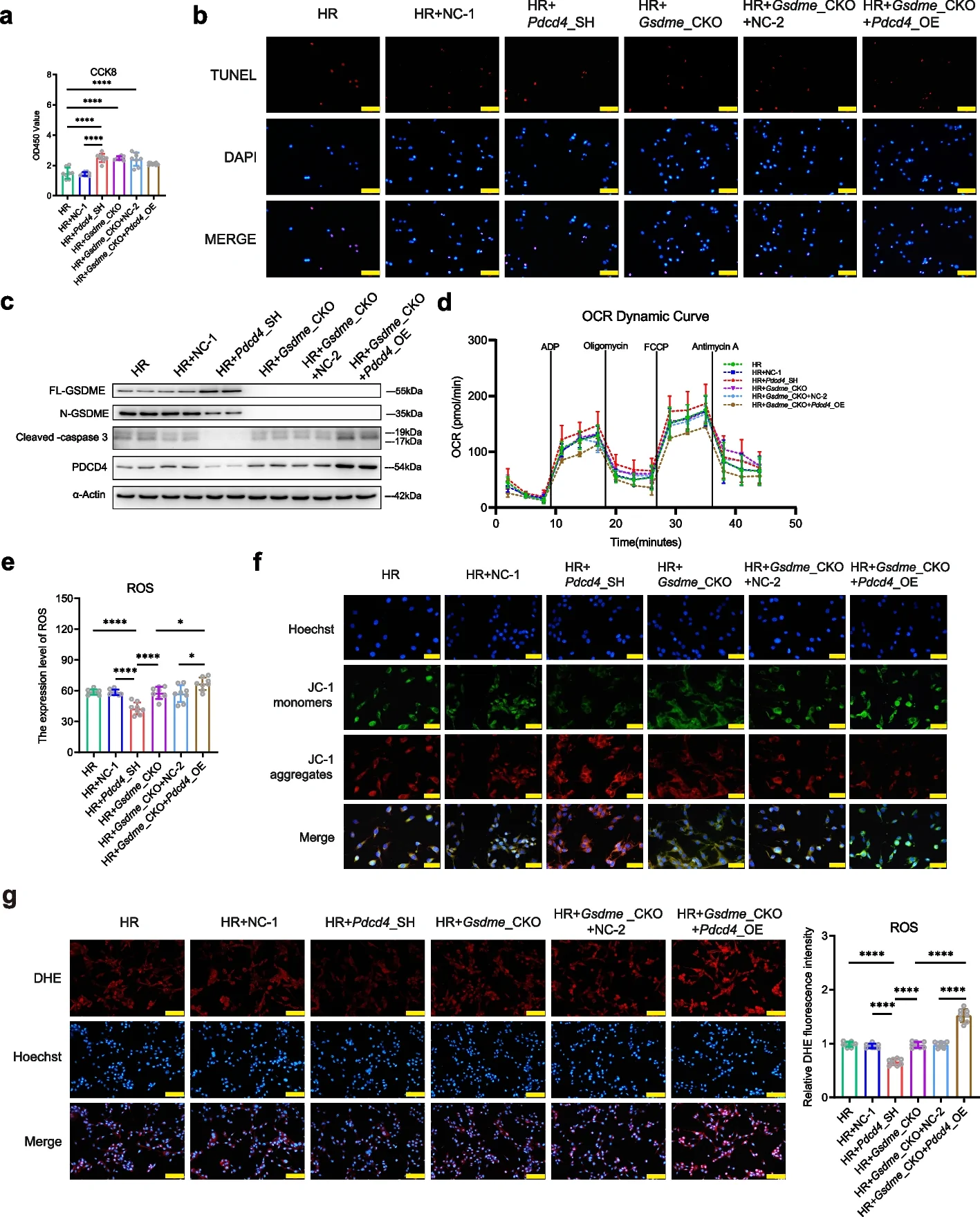
Effects of GSDME deletion and PDCD4 manipulation on hypoxia-reoxygenation-induced cardiomyocyte injury and mitochondrial function. Primary cardiomyocytes were assigned to H/R, H/R + NC-1, H/R + *Pdcd4*-SH, H/R + *Gsdme*-cKO, H/R + *Gsdme*-cKO + NC-2, or H/R + *Gsdme*-cKO + *Pdcd4*-OE groups. **a** Cell viability assessed by CCK-8. **b** Representative TUNEL staining. Scale bar, 100 µm. **c** Immunoblot analysis of FL-GSDME, N-GSDME, cleaved caspase-3, and PDCD4. **d** OCR profiles during the mitochondrial stress test. **e** Intracellular ROS levels. **f** Representative JC-1 fluorescence images showing nuclei, JC-1 monomers, JC-1 aggregates, and merged images. Scale bar, 50 µm. **g** Representative dihydroethidium (DHE) and Hoechst fluorescence images and quantification of relative DHE fluorescence intensity. For quantitative panels, data are presented as mean ± SD (*n* = 8 per group). Statistical significance was determined by one-way ANOVA followed by Tukey's multiple-comparison test. *P < 0.05, **P < 0.01, ***P < 0.001, and ****P < 0.0001; ns, not significant. cKO, conditional knockout

To examine the relationship between PDCD4-induced mitochondrial injury and GSDME-dependent cell death, we modulated PDCD4 expression in primary cardiomyocytes with or without GSDME deficiency. Under H/R conditions, GSDME-deficient cardiomyocytes showed preserved cell viability despite PDCD4 overexpression (Fig. 6a). Reduced cell injury was also observed in these cells (Fig. 6b-c; Fig. S8a-e). However, PDCD4-associated mitochondrial dysfunction and ROS accumulation persisted despite GSDME deletion (Fig. 6d-g; Fig. S8f-g). The persistence of mitochondrial abnormalities despite reduced cell injury supports placement of mitochondrial dysfunction upstream of GSDME-dependent cell death. Together, these findings support a model in which PDCD4 promotes mitochondrial injury and ROS accumulation, facilitating downstream caspase-3-mediated GSDME activation and pyroptosis. Within this model, IGFBP2-AKT signaling suppresses PDCD4 and protects mitochondria upstream of GSDME activation.

### Aerobic exercise preconditioning downregulates PDCD4 through IGFBP2/PI3K/AKT pathway to suppress GSDME-dependent Pyroptosis in vivo

**Fig. 7:**
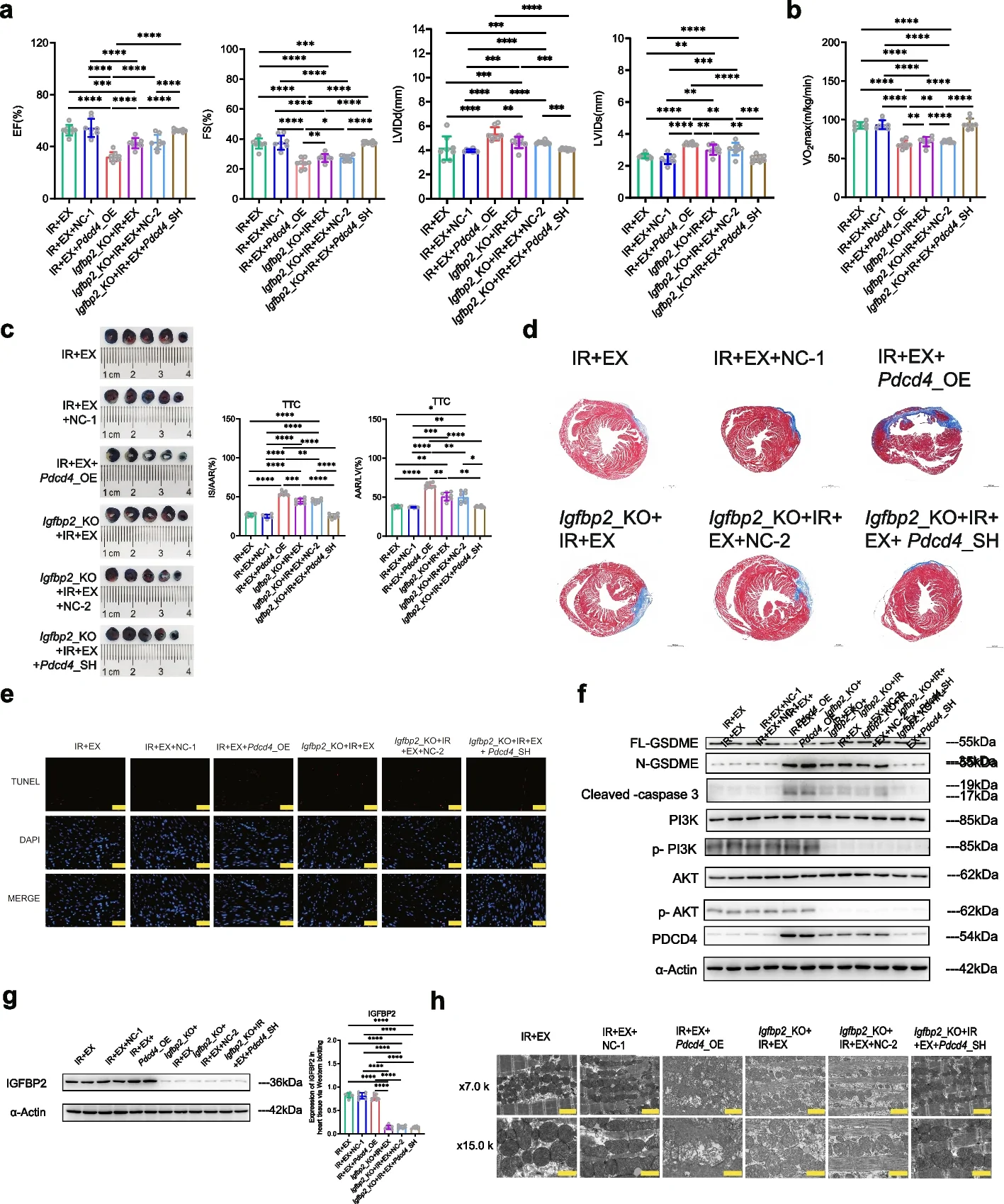
Exercise-induced IGFBP2 activates PI3K-AKT signaling and suppresses PDCD4 to protect the myocardium during ischemia–reperfusion. Mice were assigned to I/R + EX, I/R + EX + NC-1, I/R + EX + *Pdcd4*-OE, *Igfbp2*-KO + I/R + EX, *Igfbp2*-KO + I/R + EX + NC-2, or *Igfbp2*-KO + I/R + EX + *Pdcd4*-SH groups. **a** Echocardiographic measurements of EF, FS, LVIDd, and LVIDs. **b** VO₂max in the indicated groups. **c** Representative TTC-stained heart sections collected 24 h after I/R and quantification of infarct size relative to the area at risk (IS/AAR). **d** Representative Masson's trichrome staining of myocardial fibrosis 4 weeks after I/R. **e** Representative TUNEL staining of myocardial sections. Scale bar, 50 µm. **f** Immunoblot analysis of FL-GSDME, N-GSDME, cleaved caspase-3, total and phosphorylated PI3K and AKT, and PDCD4 in myocardial tissue. **g** IGFBP2 immunoblotting and densitometric quantification. (h) Representative TEM images of myocardial mitochondria. Scale bars, 2 µm (7.0 K) and 1 µm (15.0 K). For quantitative panels, data are presented as mean ± SD (*n* = 8 per group). Statistical significance was determined by one-way ANOVA followed by Tukey's multiple-comparison test. *P < 0.05, **P < 0.01, ***P < 0.001, and ****P < 0.0001; ns, not significant

To validate these regulatory mechanisms in vivo, *Igfbp2*-KO mice and WT mice were subjected to myocardial I/R injury with or without aerobic exercise preconditioning. Cardiac PDCD4 expression was modulated using AAV9 vectors. Consistent with our in vitro findings, aerobic exercise preconditioning improved cardiac function (Fig. 7a) and cardiopulmonary performance (Fig. 7b), reduced IS/AAR ratio and myocardial fibrosis (Fig. 7c-d; Fig. S8h), suppressed GSDME-dependent pyroptosis (Fig. 7e-f; Fig. S8i-j), and decreased myocardial inflammatory cytokine release, including IL-1β and IL-18 (Fig. S8k-l). Immunoblotting further showed that aerobic exercise preconditioning increased p-PI3K and p-AKT levels while decreasing cleaved GSDME, cleaved caspase-3, and PDCD4 levels; these molecular changes were largely lost in the setting of *Pdcd4* overexpression or *Igfbp2* knockout (Fig. 7f; Fig. S8j). IGFBP2 immunoblotting confirmed reduced myocardial IGFBP2 levels in *Igfbp2*-deficient mice (Fig. 7g). Aerobic exercise preconditioning also preserved mitochondrial ultrastructure (Fig. 7h; Fig. S8m). In contrast, *Pdcd4* overexpression or *Igfbp2* deficiency abrogated these protective effects, whereas *Pdcd4* knockdown in *Igfbp2*-deficient mice partially restored them. Taken together, these data support a model in which aerobic exercise preconditioning-induced IGFBP2 signals through PI3K/AKT to suppress PDCD4, preserve mitochondrial integrity, and reduce GSDME-dependent pyroptosis during myocardial I/R injury.

## Discussion

Myocardial I/R injury remains a major cause of cardiac dysfunction after acute myocardial infarction. In our previous Molecular Medicine study, exercise began 1 week after I/R and continued for 8 weeks, as illustrated in that study’s Fig. 1 [10]. In the present study, mice completed 12 weeks of exercise before I/R (Fig. 1a); we therefore use “exercise preconditioning” for the current protocol and “post-I/R exercise” for the previous protocol. Further untargeted proteomic screening of mouse myocardium identified PDCD4 and PIK3CA as candidate mediators, with decreased PDCD4 and increased PIK3CA abundance after exercise (Fig. 4b-c). The principal advance is the integration of human plasma proteomic evidence linking sustained exercise habits to higher circulating IGFBP2 with experimental validation of the IGFBP2-IGF-1R-PI3K/AKT-PDCD4 pathway. Our data support a model in which IGFBP2-IGF-1R/PI3K/AKT signaling suppresses PDCD4, thereby preserving mitochondrial integrity and limiting downstream GSDME activation. In the loss-of-function experiments, GSDME-deficient cardiomyocytes showed reduced cell injury despite PDCD4 overexpression, whereas mitochondrial dysfunction and ROS accumulation persisted. This dissociation supports positioning PDCD4-induced mitochondrial injury upstream of GSDME activation in the proposed mechanism, with GSDME acting as a downstream effector of pyroptotic cell death. These findings enrich understanding of aerobic exercise preconditioning-induced cardioprotection and highlight potential molecular targets for future intervention in I/R injury.

Pyroptosis, a pro-inflammatory form of programmed cell death, has been increasingly recognized as a critical mechanism in myocardial I/R injury [9, 24, 25]. Among the gasdermin family, GSDME is a key executor whose cleavage triggers cell membrane pore formation and the release of pro-inflammatory cytokines which aggravate tissue injury. Previous studies have linked exercise-responsive signaling pathways to cardiomyocyte survival and mitochondrial protection, including our work on IGFBP2-AKT-GSK3β-VDAC1 signaling and GSDME-dependent pyroptosis during post-I/R recovery [10, 16–18]. The present findings extend this mechanism by identifying PDCD4 suppression downstream of IGFBP2-IGF-1R-PI3K/AKT signaling and linking it to mitochondrial preservation upstream of GSDME activation. Together with the human proteomic evidence, this mechanistic extension provides the basis for the study’s novelty beyond the difference in exercise timing.

Traditionally, the cardioprotective benefits of aerobic exercise have been attributed to improvements in coronary perfusion, enhanced antioxidant capacity, modulation of autophagy, and attenuation of apoptosis [16, 26–28]. Recent study reported that moderate-intensity aerobic exercise suppresses activation of the NLRP3 inflammasome, thereby alleviating cardiomyocyte pyroptosis during I/R episodes [29]. Our current data demonstrated that aerobic exercise preconditioning activates the PI3K/AKT pathway and downregulates PDCD4, which aligns with insights from related work—Yang et al. showed that aerobic exercise ameliorated obesity-induced myocardial pyroptosis and inflammation by modulating PI3K/AKT axis [30]. Both studies reinforce the central importance of PI3K/AKT signaling in exercise-mediated regulation of myocardial pyroptosis. Our findings extend this understanding by supporting a model in which PDCD4 promotes mitochondrial injury and ROS accumulation upstream of GSDME activation, linking exercise-responsive IGFBP2 signaling to mitochondrial preservation and reduced pyroptosis.

Our findings support IGFBP2 as an important mediator contributing to exercise-induced cardioprotection and show an associated increase in circulating IGFBP2. As a binding protein of IGF-1, IGFBP2 has not been fully elucidated its role in I/R injury as IGF-1 did. Through rigorous in vivo and in vitro experiments, including *Igfbp2*-knockout models, we showed that IGFBP2 loss abolished the protective effects of aerobic exercise preconditioning observed in this mouse model, whereas IGFBP2 was required for downstream activation of IGF-1R/PI3K/AKT signaling, suppression of PDCD4 and attenuation of GSDME-dependent pyroptosis by its protective effect on mitochondria integrity. These insights position IGFBP2 not merely as a passive IGF-1 modulator but as an active player in the myocardial response to aerobic exercise preconditioning, providing a strong rationale for its consideration as a therapeutic target.

The *Igfbp2* knockout and IGF-1R-deficiency experiments support a contribution of IGFBP2-IGF-1R signaling to exercise-induced cardioprotection in the models examined. However, these findings do not establish that IGFBP2 alone is sufficient to reproduce the full cardioprotective effects of exercise. Other exercise-responsive pathways may also contribute, and their relative contributions and interactions with IGFBP2 signaling remain to be determined.

Nonetheless, this study has limitations that warrant further exploration. The human proteomic analysis included a small, male-only cohort, which limits generalization to women. Incomplete individual training records also limited assessment of exercise frequency, session duration, and relationships between training dose and proteomic responses. Global *Igfbp2* knockout demonstrates a requirement for IGFBP2 in exercise-induced protection in this model, but it does not identify the tissue of origin or distinguish the contributions of circulating and locally produced IGFBP2. The rise in plasma IGFBP2 therefore does not establish circulating IGFBP2 as the source responsible for protection. Tissue-specific deletion and selective rescue experiments are needed to resolve these contributions. TUNEL detects DNA fragmentation and is not specific for pyroptosis; it cannot distinguish pyroptosis from apoptosis or other forms of cell death. TUNEL positivity was therefore interpreted alongside GSDME cleavage, membrane-associated N-GSDME, membrane permeability measurements, and the in vitro GSDME loss-of-function findings, rather than as standalone evidence of pyroptosis. These findings do not exclude coexisting cell-death pathways. In addition, the HEK-293T reporter assay measures the activity of the specified PDCD4 promoter construct in a heterologous system and does not establish direct transcriptional regulation of endogenous PDCD4 in cardiomyocytes. Although we delineated the IGFBP2-IGF-1R-PI3K/AKT axis, the precise molecular interactions by which IGFBP2 regulates PDCD4 expression remain to be clarified. Another limitation is that TUNEL staining detects DNA fragmentation and cannot distinguish pyroptosis from other forms of cell death; therefore, our interpretation of GSDME-mediated pyroptotic cell death is supported by the combined evidence of GSDME cleavage and membrane localization, PI uptake, and LDH release. Moreover, while our findings are robust in rodents and primary cardiomyocytes, additional validation in larger animal models and clinical cohorts is necessary to ascertain translational relevance. It is also important to note that myocardial I/R injury involves multiple overlapping forms of programmed cell death, including apoptosis and necroptosis; future studies should assess whether IGFBP2 influences these pathways as well, potentially broadening its therapeutic impact. Besides, the optimal aerobic exercise preconditioning regimen required to maximally activate protective pathways and maximal achieve cardioprotection following I/R injury remains to be systematically determined in the future.

## Conclusion

This study demonstrated that long-term aerobic exercise significantly attenuates myocardial I/R injury by upregulating circulating IGFBP2. This elevation activates the IGF-1R/PI3K/AKT pathway, leading to downregulation of PDCD4 and inhibition of GSDME-dependent pyroptosis. These results not only refine our understanding of exercise-mediated cardioprotection but also highlight IGFBP2 as a promising target for developing new therapeutic strategies against myocardial I/R injury.

## Materials and methods

### Human plasma proteomics

Eligible participants were healthy adults aged 18 years or older. Through an open invitation for blood sampling, we recruited 34 men: 17 marathon runners with a history of regular aerobic exercise (exercise group) and 17 age- and health-matched men with no history of regular exercise training (control group). Individuals younger than 18 years or not meeting the health and group-specific exercise-history criteria were ineligible.

For exercise-group participants, the available training-history information indicated an average of approximately 8 years of regular aerobic exercise, a typical running volume of approximately 10 km/week, and an estimated exercise intensity of 60–70% of peak oxygen consumption (VO₂peak). Detailed individual training records were unavailable; the number of training sessions per week and duration per session could therefore not be reliably specified.

Baseline height, weight, age, and cardiopulmonary exercise testing (CPET) results (VO₂max and anaerobic threshold) were collected at recruitment. BMI was calculated for each participant as body weight (kg) divided by height squared (m^2^). In the exercise group, blood samples were collected at baseline, one day before exercise intervention (T1), within immediately after aerobic exercise (T2), and 2 weeks after aerobic exercise (T3). The demographic characteristics of the participants are provided in Supplementary Material and Table S1. Participants in the control group underwent blood sampling at T0 only. Plasma samples were processed for untargeted proteomic analysis. The mass spectrometryproteomics data have been deposited to the ProteomeXchange Consortium(https://proteomecentral.proteomexchange.org) via the iProX partner repository [31, 32] with the datasetidentifier PXD082464.

### Animals and experimental design

Male C57BL/6 J mice aged 6–8 weeks, Myh6-Cre mice, *Igfbp2* knockout mice, and Igf-1r conditional floxed mice were obtained from Cyagen Biosciences Inc. (Suzhou, China). Igf-1r conditional knockout mice were generated by crossing Igf-1r^fl/fl^ mice with Myh6-Cre mice to obtain Myh6-Cre/Igf-1r^fl/fl^ mice. Male littermates carrying Igf-1r^fl/fl^ or Myh6-Cre alone were used as controls where appropriate. Mice were randomly assigned to 11 experimental groups, with 20 mice per group: control, exercise, I/R, I/R + exercise, Igfbp2 knockout + I/R, Igfbp2 knockout + I/R + exercise, Igf-1r conditional knockout + I/R + exercise, I/R + exercise + AAV9-Pdcd4 overexpression, I/R + exercise + AAV9 negative control, Igfbp2 knockout + I/R + exercise + AAV9-shPdcd4, and Igfbp2 knockout + I/R + exercise + AAV9 negative control. Investigators were blinded to group allocation during outcome assessment whenever feasible.

### Aerobic exercise intervention and mice heart tissue proteomics

Mice in the exercise groups completed a 12-week treadmill-based aerobic exercise programme before sham surgery or myocardial I/R, with one 55-min session per day from Monday to Friday (Fig. 1a). This protocol is referred to throughout as exercise preconditioning. Maximal oxygen consumption (VO₂_max_) was assessed every Saturday using a gas metabolism monitoring system and modular treadmill system (Columbus Instruments, Columbus, OH, USA). Exercise intensity for the following week was adjusted according to the preceding VO_₂max_ measurement and maintained at approximately 50% VO₂_max_. Non-exercised control mice were placed on the treadmill for the same duration without running. Exercise capacity was assessed using a graded incremental treadmill protocol consisting of three stages: 0° incline at 8.2 m/min for 15 min, 5° incline at 15 m/min for 15 min, and 10° incline at 19.3 m/min for 15 min. Exhaustion was defined as the inability to maintain running despite stimulation and speed reduction, accompanied by persistent contact with the rear of the treadmill and failure to resume running. The heart tissue of mice with or without aerobic exercise intervention were processed for untargeted proteomic analysis. The mass spectrometry mice heart tissue proteomics data have been deposited to the ProteomeXchange Consortium (https://proteomecentral.proteomexchange.org) via the iProX partner repository [31, 32] with the dataset identifier PXD082393.

### Myocardial ischemia/reperfusion model

After the 12-week exercise or sedentary period, myocardial I/R was induced in male mice by transient ligation of the left anterior descending coronary artery (Fig. 1a). Prior to surgery, the mice were fasted from food and water for 8 h. Anesthesia was induced via a single intraperitoneal injection of ketamine (40 mg/kg) and xylazine (5 mg/kg). After left thoracotomy, the left anterior descending artery was ligated with an 8–0 suture over a PE-10 polyethylene tube. Successful ischemia was confirmed by pallor of the anterior left ventricular wall. After 30 min of ischemia, the tube was removed to allow reperfusion, which was confirmed by restoration of myocardial colour. The chest was then closed in layers. At the end of the experiments, mice were euthanized with intravenous sodium pentobarbital (800 mg/kg). Postoperative analgesia was administered using buprenorphine at a dose of 0.1 mg/kg via subcutaneous injection (s.c.).

### Primary cardiomyocyte isolation and hypoxia/reoxygenation model

Primary neonatal mouse cardiomyocytes were isolated from 1–3-day-old C57BL/6 J mice (anaesthetized with 2% isoflurane via inhalation). Ventricular apical tissues were minced and sequentially digested with trypsin and collagenase II. Cell suspensions were filtered, centrifuged, and pre-plated to reduce non-cardiomyocyte contamination. Cardiomyocytes were cultured in DMEM/Medium 199 (Sigma-Aldrich, St. Louis, USA) supplemented with 10% fetal bovine serum, BrdU, penicillin, and streptomycin at 37 °C in 5% CO₂. To establish the hypoxia/reoxygenation model, cardiomyocytes were exposed to hypoxia in a chamber containing 95% N₂ and 5% CO₂ for 4 h, followed by reoxygenation under normoxic conditions for 2 h. Where indicated, cells were treated with 250 ng/mL of recombinant IGFBP2 protein (Cat. No. 84092–1-RR, Proteintech, Wuhan, China), 25 μM of the PI3K inhibitor LY294002 (Selleckchem, Shanghai, China), or 5 μM of the PI3K agonist 740 Y-P (Selleckchem, Shanghai, China) before hypoxia/reoxygenation. Cell viability, protein expression, mitochondrial function, oxidative stress, and inflammatory markers were assessed after treatment.

### Viral vector administration and lentiviral transduction

For in vivo modulation of *Pdcd4* expression, Mice in the Pdcd4 overexpression group were intravenous injected with 200 μL of cardiomyocyte-specific AAV9-m-Pdcd4 (Vector Biolabs, Philadelphia, USA) at a titer of 1 × 10^12^ μg/mL (total dose: 2 × 10^11^ vg per mouse). Similarly, mice in the Pdcd4 knockdown group were injected with cardiomyocyte-specific AAV9-shPdcd4 (GeneCopoeia, Rockville, USA) at the same titer and dose. Corresponding negative control groups were injected with empty AAV9 vectors (Beyotime, Shanghai, China). For in vitro experiments, neonatal mouse cardiomyocytes were seeded into 6-well plates and cultured until reaching 70–80% confluence. *Pdcd4* overexpression lentivirus (pHBLPm001435-plasmid, Hanheng Bioengineering, Shanghai, China) and shPdcd4 lentivirus (L23156, Beyotime, Shanghai, China) were both diluted to 2 × 10^8^ TU/ml using Opti-MEM medium. Polybrene was added at a final concentration of 8 μg/mL to enhance transduction efficiency. The viral mixtures were gently added to the culture medium and incubated with the cells for 68 h. After infection, the viral supernatant was removed and replaced with fresh complete DMEM medium containing 10% fetal bovine serum (FBS), and the cells were cultured for another 48 h before being harvested for subsequent assays. For the Gsdme_CKO group, primary cardiomyocytes isolated from Gsdme^fl/fl^ mice were infected with Lenti-CMV-Cre lentivirus (plasmid #154877, Addgene, Watertown, MA, USA). The corresponding negative control cells were infected with an equivalent amount of empty control lentivirus (Beyotime, Shanghai, China).

### Echocardiography

Cardiac function was evaluated by transthoracic echocardiography at the first and fourth weeks after I/R surgery. Mice were anaesthetized with isoflurane and examined using a Vevo 2100 high-resolution ultrasound system equipped with a 30-MHz transducer (VisualSonics, Toronto, Canada). Heart rate and body temperature were monitored and controlled during image acquisition; other physiological parameters were not monitored. Detailed numerical monitoring records were not retained. B-mode and M-mode images were acquired from long-axis and short-axis views. Left ventricular ejection fraction, fractional shortening, left ventricular internal diameter in diastole, and left ventricular internal diameter in systole were measured.

### Histological and ultrastructural analysis

Myocardial IS/AAR ratio was assessed by 1% 2,3,5-triphenyltetrazolium chloride staining after Evans Blue (IE0280, Solarbio, China) injection (200 μl per mouse, 1% PBS solution). Hearts were sectioned into 1–2-mm transverse slices, incubated with TTC at 37 °C, fixed in 4% paraformaldehyde, photographed, and analysed using Image-Pro Plus 6.0. Myocardial fibrosis was evaluated using Masson’s trichrome staining. Paraffin-embedded heart sections were stained using a commercial Masson kit, and fibrotic area was quantified as the percentage of fibrotic area relative to total myocardial area using ImageJ. DNA fragmentation was assessed using TUNEL staining in myocardial tissue sections and cultured cardiomyocytes according to the manufacturer’s protocol. TUNEL was used as a nonspecific indicator of cell death and was not considered a pyroptosis-specific assay. Nuclei were counterstained with DAPI, and TUNEL-positive cells were quantified using ImageJ. Mitochondrial ultrastructure was examined by transmission electron microscopy. Myocardial tissues or cardiomyocytes were fixed, dehydrated, embedded, sectioned, and stained with uranyl acetate and lead citrate. Mitochondrial morphology, area, and number were analyzed using ImageJ.

### Mitochondrial membrane potential, ROS production, and cell viability

Mitochondrial membrane potential was evaluated using JC-1 staining. After treatment, cardiomyocytes were incubated with JC-1 working solution, and fluorescence was assessed by fluorescence microscopy. Intracellular ROS levels were measured using a Reactive Oxygen Species Fluorometric Assay Kit (Green) based on DCFH-DA (Elabscience, Wuhan, China; Cat. No. E-BC-K138-F). Cardiomyocytes were incubated with 10 μM DCFH-DA at 37 °C for 30 min in the dark and washed two to three times with serum-free medium. DCF fluorescence was visualized by fluorescence microscopy and quantified using a fluorescence microplate reader.

DHE fluorescence was detected using a DHE-based ROS assay kit (Servicebio, Wuhan, China; Cat. No. G1746) according to the manufacturer’s instructions. Images were acquired using identical fluorescence microscopy settings across groups, and the mean fluorescence intensity was quantified. Cell viability was assessed using the Cell Counting Kit-8 assay (Servicebio, Wuhan, China; G4103). Cardiomyocytes were seeded into 96-well plates, treated as indicated, incubated with CCK-8 reagent, and absorbance was measured at 450 nm using a microplate reader. Statistical significance was determined by one-way or two-way ANOVA followed by Tukey's multiple-comparison test.

### Western blotting, ELISA, and co-immunoprecipitation

Total protein was extracted from mouse heart tissues or cardiomyocytes using a total protein extraction kit (G3741, Servicebio, Wuhan, China), and protein concentration was determined using a BCA assay (G2026, Servicebio, Wuhan, China). Equal amounts of protein were mixed with 2 × loading buffer (Servicebio, Wuhan, China) and boiled. Proteins were separated by SDS-PAGE gel, transferred onto PVDF membranes. The membranes were blocked with 5% BSA and then incubated overnight at 4 °C with the following primary antibodies: N-GSDME (ab215191, 1:2000, Abcam, Cambridge, UK), cleaved caspase-3 (82707–13-RR, 1:10000, Proteintech, Shanghai, China), cleaved caspase-1 (YM9369, 1:2000, IMMUNOWAY, CA, USA), NLRP3 (YM8024, 1:500, IMMUNOWAY, CA, USA), N-GSDMD (YM4266, 1:500, IMMUNOWAY, CA, USA), PI3K (ab191606, 1:1000, Abcam, Cambridge, UK), AKT (10176–2-AP, 1:10000, Proteintech, Wuhan, China), phospho-PI3K (17366 T, 1:1000, Cell Signaling, Danvers, USA), phospho-AKT (AP0140, 1:1000, ABclonal, Wuhan, China), IGFBP2 (ab188200, 1:5000, Abcam, Cambridge, UK), IGF-1R (20254–1-AP, 1:2000, Proteintech, Wuhan, China), and PDCD4 (12587–1-AP, 1:2000, Proteintech, Shanghai, China) After incubation with HRP-conjugated secondary antibodies, signals were visualized using enhanced chemiluminescence and quantified using ImageJ. Levels of IGFBP2 (KE10151, Proteintech, Wuhan, China), LDH (ab102526, Abcam, Cambridge, UK), IL-1β (ab197742, Abcam, Cambridge, UK), and IL-18 (ab216165, Abcam, Cambridge, UK) in tissue or cell samples were measured using commercial ELISA kits according to the manufacturers’ instructions. Absorbance was measured at 450 nm. For co-immunoprecipitation, heart tissues or cardiomyocytes were lysed, and protein extracts were incubated with anti-IGF-1R (20254–1-AP, Proteintech, Wuhan, China) or anti-IGFBP2 (ab188200, Abcam, Cambridge, UK) antibodies followed by Protein A/G magnetic beads (Bimake, Houston, USA). Immunoprecipitated proteins were eluted and analysed by western blotting to assess the interaction between IGFBP2 and IGF-1R. Statistical significance was determined by one-way or two-way ANOVA followed by Tukey's multiple-comparison test.

### PDCD4 promoter-luciferase reporter assay in HEK-293 T cells

A PDCD4 promoter-luciferase reporter assay was performed in HEK-293 T cells as a heterologous cell system. Cells were transfected with a PDCD4 promoter-driven firefly luciferase reporter construct (pGL3-PDCD4 promoter) and subsequently treated with 5 μM 740 Y-P or the corresponding solution control (CON). Luciferase activity was measured using the Dual-Lucy Assay Kit (Solarbio, Beijing, China; catalog no. D0011) according to the manufacturer’s instructions. The corresponding relative light unit values were measured immediately after each addition using microplate reader in luminescence mode. After background subtraction, firefly luciferase activity was normalized to Renilla luciferase activity, and the resulting ratios were expressed relative to those of the CON group.

### Immunofluorescence

Primary cardiomyocytes cultured on coverslips were subjected to the indicated treatments, washed with phosphate-buffered saline, fixed with paraformaldehyde, and blocked with bovine serum albumin. Cells were incubated overnight at 4 °C with an antibody recognizing the N-terminal region of GSDME (ab215191, Abcam), followed by incubation with an appropriate fluorophore-conjugated secondary antibody in the dark. Cell membranes were labeled with wheat germ agglutinin (WGA), and nuclei were counterstained with Hoechst. WGA staining was used to delineate cardiomyocyte membranes, and the background-subtracted mean fluorescence intensity of membrane-associated N-GSDME was quantified using ImageJ software. Randomly selected, nonoverlapping fields were analyzed for each independent experiment.

### Seahorse

Mitochondrial respiration in primary neonatal mouse cardiomyocytes was assessed using a Seahorse XF Extracellular Flux Analyzer (Agilent Technologies, Santa Clara, CA, USA). Primary ventricular cardiomyocytes were isolated from 1–3-day-old C57BL/6 J mice. Briefly, the hearts were rapidly excised under sterile conditions, minced, and subjected to serial digestion with type II collagenase and trypsin. The resulting cell suspensions were collected, filtered, centrifuged, and subjected to differential preplating to reduce fibroblast contamination. Purified cardiomyocytes were then seeded at a density of 3–5 × 10^4^ cells per well in Seahorse XF96 cell culture microplates precoated with type I collagen and cultured overnight in complete medium. On the day of the assay, cells were approximately 80%−90% confluent, firmly attached, and distributed as a uniform monolayer. Before analysis, the culture medium was replaced with prewarmed Seahorse XF base medium supplemented with 10 mmol/L glucose, 1 mmol/L sodium pyruvate, and 2 mmol/L glutamine. Cells were equilibrated in a non-CO₂ incubator at 37 °C for 45–60 min. Mitochondrial respiratory function was evaluated using the Seahorse XF Cell Mito Stress Test Kit (Agilent Technologies; Cat. No. 103015–100) according to the manufacturer’s instructions. The oxygen consumption rate (OCR) was monitored in real time. After recording basal OCR, oligomycin, carbonyl cyanide 4-(trifluoromethoxy)phenylhydrazone (FCCP), and rotenone/antimycin A were sequentially injected to inhibit ATP synthase, uncouple mitochondrial oxidative phosphorylation, and inhibit mitochondrial complexes I and III, respectively. Changes in OCR following these injections were used to calculate ATP-linked respiration, maximal respiratory capacity, spare respiratory capacity, and nonmitochondrial oxygen consumption. After the assay, cells in each well were lysed, and total protein content was determined using a bicinchoninic acid assay. OCR values were normalized to the total protein content of the corresponding wells. Five or six technical replicate wells were included for each experimental condition, and the experiments were independently repeated at least three times.

### Statistical analysis

Data are presented as mean ± standard deviation (SD), unless otherwise indicated. Baseline characteristics of the independent human control and exercise groups were compared using two-sided unpaired Student’s t-tests. The log₂-transformed protein abundances in Fig. 4c were compared using unpaired Student’s t-tests.Experiments with two fully crossed factors were analysed using ordinary two-way analysis of variance (ANOVA), with both main effects and their interaction included in the model, followed by Tukey’s multiple-comparison test. The related statistics are provided in Table S2.

For the longitudinal IGFBP2 measurements in Fig. 2e-f, the Geisser-Greenhouse correction was applied to the omnibus ANOVA, followed by Tukey-adjusted pairwise comparisons. Comparisons between the control group and each exercise time point were performed using two-sided Welch’s t-tests, with Holm adjustment across the three comparisons. Statistical significance was defined as *P* < 0.05. Multiplicity-adjusted P values were used for the multiple comparisons described above.

## Supplementary Information

Supplementary Material 1

## Data Availability

Additional data and materials supporting the findings of this study are available from the corresponding author upon reasonable request. The mass spectrometryproteomics data have been deposited to the ProteomeXchange Consortium (https://proteomecentral.proteomexchange.org) via the iProX partner repository [31, 32] with the datasetidentifier PXD082464 and PXD082393.

## Abbreviations

AAV9: Adeno-associated virus serotype 9
ADV: Adenoviral vector
AKT: Protein kinase B
ANOVA: Analysis of variance
ATP: Adenosine triphosphate
BCA: Bicinchoninic acid
BMI: Body mass index
BrdU: 5-Bromo-2′-deoxyuridine
BSA: Bovine serum albumin
C: Control group
CCK-8: Cell Counting Kit-8
cKO/CKO: Conditional knockout
CMV: Cytomegalovirus
CON: Control
CPET: Cardiopulmonary exercise testing
DAPI: 4′,6-Diamidino-2-phenylindole
DCF: 2′,7′-Dichlorofluorescein
DCFH-DA: 2′,7′-Dichlorodihydrofluorescein diacetate
DHE: Dihydroethidium
DMEM: Dulbecco’s modified Eagle’s medium
DNA: Deoxyribonucleic acid
EF: Ejection fraction
ELISA: Enzyme-linked immunosorbent assay
EX: Exercise
FBS: Fetal bovine serum
FCCP: Carbonyl cyanide 4-(trifluoromethoxy)phenylhydrazone
FL-GSDME: Full-length gasdermin E
FS: Fractional shortening
GSDMD: Gasdermin D
GSDME: Gasdermin E
GSK3β: Glycogen synthase kinase 3 beta
HEK-293T: Human embryonic kidney 293T cells
H/R: Hypoxia/reoxygenation
HRP: Horseradish peroxidase
IGF-1: Insulin-like growth factor 1
IGF-1R: Insulin-like growth factor 1 receptor
IGFBP2: Insulin-like growth factor-binding protein 2
IL-18: Interleukin-18
IL-1β: Interleukin-1 beta
I/R: Ischemia/reperfusion
IS/AAR: Infarct size relative to the area at risk
JC-1: 5,5′,6,6′-Tetrachloro-1,1′,3,3′-tetraethylbenzimidazolylcarbocyanine iodide
KO: Knockout
LDH: Lactate dehydrogenase
LVIDd: Left ventricular internal diameter at end-diastole
LVIDs: Left ventricular internal diameter at end-systole
Myh6: Myosin heavy chain 6
NC: Matched negative control
N-GSDMD: N-terminal fragment of gasdermin D
N-GSDME: N-terminal fragment of gasdermin E
NLRP3: NLR family pyrin domain-containing protein 3
ns: Not significant
OCR: Oxygen consumption rate
OE: Overexpression
OXPHOS: Oxidative phosphorylation
p-AKT: Phosphorylated AKT
PBS: Phosphate-buffered saline
PCD: Programmed cell death
PDCD4: Programmed cell death protein 4
PI: Propidium iodide
PI3K: Phosphoinositide 3-kinase
PIK3CA: Phosphatidylinositol-4,5-bisphosphate 3-kinase catalytic subunit alpha
p-PI3K: Phosphorylated PI3K
PVDF: Polyvinylidene difluoride
ROS: Reactive oxygen species
SD: Standard deviation
SDS-PAGE: Sodium dodecyl sulfate–polyacrylamide gel electrophoresis
SH: Knockdown (experimental group designation)
TEM: Transmission electron microscopy
TTC: 2,3,5-Triphenyltetrazolium chloride
TUNEL: Terminal deoxynucleotidyl transferase dUTP nick-end labeling
VDAC1: Voltage-dependent anion channel 1
VO₂max: Maximal oxygen consumption
VO₂peak: Peak oxygen consumption
WGA: Wheat germ agglutinin
WT: Wild type
