# Supplementary material for: Aerobic exercise preconditioning inhibits GSDME-dependent pyroptosis in myocardial ischemia–reperfusion injury through IGFBP2-AKT-PDCD4 signaling
Source: Mol Biomed. 2026 Nov 5;7:239. doi: 10.1186/s43556-026-00633-y (PMC13740222; doi:10.1186/s43556-026-00633-y)
Supplement: Supplementary file 1 [file 43556_2026_633_MOESM1_ESM.docx]

Supplementary material

Supplementary Figure S1

Supplementary Figure 1. Additional analyses of myocardial fibrosis, DNA fragmentation, and inflammatory signaling after exercise preconditioning.

Myocardial measurements in control (CON) and ischemia–reperfusion (I/R) mice with or without 12 weeks of exercise preconditioning (EX), corresponding to Figure 1. (a) Quantification of myocardial fibrosis corresponding to the Masson's trichrome staining in Figure 1e. (b) Quantification of terminal deoxynucleotidyl transferase dUTP nick-end labeling (TUNEL)-positive myocardial cells, indicating DNA fragmentation, corresponding to Figure 1f. (c) Densitometric quantification of cleaved caspase-3, N-terminal GSDME (N-GSDME), cleaved caspase-1, N-terminal GSDMD (N-GSDMD), and NLRP3 in myocardial tissue. (d, e) Myocardial interleukin-1β (IL-1β; d) and interleukin-18 (IL-18; e) levels in the indicated groups.

Data are presented as mean ± SD. Statistical significance was determined by two-way ANOVA followed by Tukey's multiple-comparison test. *****P* < 0.0001.

Supplementary Figure S2

Supplementary Figure 2. IGFBP2-associated protection in mouse myocardium and hypoxia–reoxygenation-treated cardiomyocytes.

Panels a–d show myocardial measurements in I/R, *Igfbp2*-KO + I/R, I/R + EX, and *Igfbp2*-KO + I/R + EX mice, corresponding to Figure 3. Panels e–l show primary cardiomyocytes with wild-type (WT) or conditionally deleted *Gsdme* (*Gsdme*-cKO) subjected to hypoxia–reoxygenation (H/R), with or without IGFBP2 pretreatment. (a) Quantification of myocardial fibrosis corresponding to the Masson's trichrome staining in Figure 3d. (b) Quantification of TUNEL-positive myocardial cells, indicating DNA fragmentation, corresponding to Figure 3e. (c, d) Myocardial IL-1β (c) and IL-18 (d) levels. (e) Cell viability assessed using the Cell Counting Kit-8 (CCK-8) assay. (f) Representative TUNEL staining with DAPI counterstaining. Scale bar, 100 µm. (g) Quantification of TUNEL-positive cardiomyocytes corresponding to panel f. (h) Immunoblot analysis of N-GSDME, cleaved caspase-3, total and phosphorylated PI3K and AKT, and PDCD4; α-actin was used as a loading control. (i) Densitometric quantification of p-AKT, N-GSDME, cleaved caspase-3, p-PI3K, and PDCD4 corresponding to panel h. (j–l) IL-1β (j), IL-18 (k), and lactate dehydrogenase (LDH; l) release from cardiomyocytes.

Data are presented as mean ± SD; *n* = 8 per group for panels e and g. Statistical significance was determined by two-way ANOVA followed by Tukey's multiple-comparison test. *****P* < 0.0001. I/R, ischemia–reperfusion; EX, exercise preconditioning; KO, knockout; cKO, conditional knockout.

Supplementary Figure S3

Supplementary Figure 3. Membrane injury and mitochondrial responses to IGFBP2 in hypoxia–reoxygenation-treated cardiomyocytes.

Panels a–c show control (CON), CON + IGFBP2, H/R, and H/R + IGFBP2 primary cardiomyocytes. Panels d–h show wild-type (WT) and *Gsdme*-deficient (*Gsdme*-cKO) cardiomyocytes subjected to H/R with or without IGFBP2 pretreatment. (a) Representative N-GSDME immunofluorescence with wheat-germ agglutinin (WGA) membrane labeling and Hoechst nuclear counterstaining. (b) Quantification of membrane-associated N-GSDME fluorescence corresponding to panel a. (c) Representative propidium iodide (PI) and Hoechst staining and quantification of PI-positive cardiomyocytes. (d) Oxygen consumption rate (OCR) profiles in the indicated groups. (e) Quantification of oxidative-phosphorylation-associated OCR corresponding to panel d. (f) Intracellular reactive oxygen species (ROS) levels. (g) Representative JC-1 fluorescence images showing Hoechst-stained nuclei, JC-1 monomers, JC-1 aggregates, and merged images. Scale bar, 50 µm. (h) Quantification of JC-1 monomer and aggregate fluorescence corresponding to panel g.

Data are presented as mean ± SD; *n* = 8 per group for panels c, d, and f. Statistical significance was determined by two-way ANOVA followed by Tukey's multiple-comparison test. ****P* < 0.001 and *****P* < 0.0001. H/R, hypoxia–reoxygenation; cKO, conditional knockout.

Supplementary Figure S4

Supplementary Figure 4. IGF-1R-associated exercise-induced cardioprotection in vivo.

Mice were assigned to I/R, I/R + EX, *Igf1r*-cKO + I/R + EX, or *Igfbp2*-KO + I/R + EX groups. (a) Circulating IGFBP2 concentrations measured by ELISA and immunoblot analysis of cardiac IGFBP2 and IGF-1R, with α-actin as the loading control. (b) Reciprocal co-immunoprecipitation analysis of the IGFBP2–IGF-1R interaction, with IgG and input controls. (c) Echocardiographic measurements of ejection fraction (EF), fractional shortening (FS), left ventricular internal diameter at end-diastole (LVIDd), and left ventricular internal diameter at end-systole (LVIDs). (d) Maximal oxygen consumption (VO₂max). (e) Representative TTC-stained heart sections. (f) Infarct size relative to the area at risk (IS/AAR) and area at risk relative to the left ventricle (AAR/LV), corresponding to panel e. (g) Representative Masson's trichrome staining of myocardial sections. Scale bar, 1000 µm. (h) Quantification of myocardial fibrosis corresponding to panel g. (i) Representative TUNEL staining with DAPI counterstaining in myocardial sections. Scale bar, 100 µm. (j) Quantification of TUNEL-positive myocardial cells corresponding to panel i. TUNEL positivity indicates DNA fragmentation and is not specific for pyroptosis.

Quantitative data are presented as mean ± SD; *n* = 8 per group for the quantitative measurements in panels a, c, and d. Quantitative panels were analyzed by one-way ANOVA followed by Tukey's multiple-comparison test. **P* < 0.05, ***P* < 0.01, ****P* < 0.001, and *****P* < 0.0001. I/R, ischemia–reperfusion; EX, exercise preconditioning; cKO, conditional knockout; KO, knockout; TTC, 2,3,5-triphenyltetrazolium chloride.

Supplementary Figure S5

Supplementary Figure 5. IGF-1R-associated PI3K/AKT signaling, inflammatory responses, and PDCD4 promoter reporter activity.

Panels a–d show myocardial measurements in I/R, I/R + EX, *Igf1r*-cKO + I/R + EX, and *Igfbp2*-KO + I/R + EX mice, corresponding to Supplementary Figure 4. (a, b) Myocardial IL-1β (a) and IL-18 (b) levels. (c) Immunoblot analysis of N-terminal GSDME (N-GSDME), cleaved caspase-3, total and phosphorylated PI3K and AKT, and PDCD4, with α-actin as the loading control. (d) Densitometric quantification of p-AKT, p-PI3K, N-GSDME, PDCD4, and cleaved caspase-3 corresponding to panel c. (e) Immunoblot analysis of total and phosphorylated PI3K and AKT, with densitometric quantification of p-AKT and p-PI3K, in wild-type (WT) and *Igf1r*-deficient cardiomyocytes subjected to H/R with or without IGFBP2. (f) Relative PDCD4 promoter–driven luciferase activity in control (CON) and PI3K-agonist-treated HEK293T cells, a heterologous cell line, transfected with the pGL3-PDCD4 promoter firefly luciferase reporter construct. Firefly luciferase activity was normalized to Renilla luciferase activity and expressed relative to CON.

Data are presented as mean ± SD; *n* = 8 per group for the quantitative measurements in panel e. Panels a, b, and d were analyzed by one-way ANOVA, and panel e was analyzed by two-way ANOVA, followed by Tukey's multiple-comparison test. Statistical significance for panel f is indicated in the panel. **P* < 0.05, ***P* < 0.01, ****P* < 0.001, and *****P* < 0.0001. I/R, ischemia–reperfusion; H/R, hypoxia–reoxygenation; EX, exercise preconditioning; cKO, conditional knockout; KO, knockout.

Supplementary Figure S6

Supplementary Figure 6. Pharmacological modulation of PI3K signaling in H/R-treated cardiomyocytes.

Cardiomyocytes were subjected to H/R with the treatments indicated in the panels. (a) Cell viability assessed using the Cell Counting Kit-8 (CCK-8) assay. (b) Representative TUNEL staining with DAPI counterstaining. Scale bar, 100 µm. (c) Quantification of TUNEL-positive cardiomyocytes corresponding to panel b. TUNEL positivity indicates DNA fragmentation and is not specific for pyroptosis. (d) Immunoblot analysis of N-terminal GSDME (N-GSDME), cleaved caspase-3, total and phosphorylated PI3K and AKT, and PDCD4, with α-actin as the loading control. (e) Densitometric quantification of p-AKT, p-PI3K, cleaved caspase-3, N-GSDME, and PDCD4 corresponding to panel d. (f–h) IL-1β (f), IL-18 (g), and lactate dehydrogenase (LDH; h) release in the indicated groups.

Quantitative data are presented as mean ± SD; *n* = 8 per group for panel a. Quantitative panels were analyzed by one-way ANOVA followed by Tukey's multiple-comparison test. **P* < 0.05, ***P* < 0.01, ****P* < 0.001, and *****P* < 0.0001. H/R, hypoxia–reoxygenation.

Supplementary Figure S7

Supplementary Figure 7. Mitochondrial responses to PI3K modulation and supporting analyses of PDCD4 manipulation.

Panels a–e show cardiomyocytes assigned to H/R, H/R + IGFBP2, H/R + IGFBP2 + the PI3K inhibitor LY294002 (25 µM), or H/R + the PI3K agonist 740 Y-P (5 µM), corresponding to the pharmacological experiments in Supplementary Figure 6a–e. Panels f–k show the PDCD4-manipulation experiments in Figure 5, comprising H/R, H/R + IGFBP2, H/R + NC-1, H/R + *Pdcd4*-SH, H/R + IGFBP2 + NC-2, and H/R + IGFBP2 + *Pdcd4*-OE groups. (a) Oxygen consumption rate (OCR) profiles in the indicated pharmacological treatment groups. (b) Quantification of oxidative-phosphorylation-associated OCR corresponding to panel a. (c) Intracellular reactive oxygen species (ROS) levels. (d) Representative JC-1 fluorescence images showing Hoechst-stained nuclei, JC-1 monomers, JC-1 aggregates, and merged images. (e) Quantification of JC-1 monomer fluorescence corresponding to panel d. (f) Quantification of TUNEL-positive cardiomyocytes, indicating DNA fragmentation, corresponding to Figure 5b. (g) Quantification of oxidative-phosphorylation-associated OCR corresponding to Figure 5d. (h) Quantification of JC-1 aggregate and monomer fluorescence corresponding to Figure 5f. (i–k) IL-1β (i), IL-18 (j), and lactate dehydrogenase (LDH; k) release from cardiomyocytes in the PDCD4-manipulation groups.

Data are presented as mean ± SD; *n* = 8 per group for panels a and c. Quantitative panels were analyzed by one-way ANOVA followed by Tukey's multiple-comparison test. **P* < 0.05, ***P* < 0.01, ****P* < 0.001, and *****P* < 0.0001. H/R, hypoxia–reoxygenation; NC, matched negative control; SH, knockdown; OE, overexpression.

Supplementary Figure S8

Supplementary Figure 8. GSDME deficiency and PDCD4 manipulation in cardiomyocytes and mouse myocardium.

Panels a–g show the cardiomyocyte experiments in Figure 6: H/R, H/R + NC-1, and H/R + *Pdcd4*-SH in wild-type (WT) cardiomyocytes, together with H/R, H/R + NC-2, and H/R + *Pdcd4*-OE in *Gsdme*-deficient cardiomyocytes. Panels h–m show myocardial measurements in the Figure 7 groups: I/R + EX, I/R + EX + NC-1, I/R + EX + *Pdcd4*-OE, *Igfbp2*-KO + I/R + EX, *Igfbp2*-KO + I/R + EX + NC-2, and *Igfbp2*-KO + I/R + EX + *Pdcd4*-SH. (a) Quantification of TUNEL-positive cardiomyocytes, indicating DNA fragmentation, corresponding to Figure 6b. (b) Densitometric quantification of full-length GSDME (FL-GSDME), N-terminal GSDME (N-GSDME), the N-GSDME/FL-GSDME ratio, cleaved caspase-3, and PDCD4 corresponding to Figure 6c. (c–e) IL-1β (c), IL-18 (d), and lactate dehydrogenase (LDH; e) release from cardiomyocytes. (f) Quantification of oxidative-phosphorylation-associated oxygen consumption rate (OCR) corresponding to Figure 6d. (g) Quantification of JC-1 aggregate and monomer fluorescence corresponding to Figure 6f. (h) Quantification of myocardial fibrosis corresponding to the Masson's trichrome staining in Figure 7d. (i) Quantification of TUNEL-positive myocardial cells, indicating DNA fragmentation, corresponding to Figure 7e. (j) Densitometric quantification of p-AKT, p-PI3K, PDCD4, cleaved caspase-3, FL-GSDME, N-GSDME, and the N-GSDME/FL-GSDME ratio corresponding to Figure 7f. (k, l) Myocardial IL-1β (k) and IL-18 (l) levels. (m) Quantification of individual mitochondrial area corresponding to the transmission electron microscopy images in Figure 7h.

Data are presented as mean ± SD. Quantitative panels were analyzed by one-way ANOVA followed by Tukey's multiple-comparison test. **P* < 0.05, ***P* < 0.01, ****P* < 0.001, and *****P* < 0.0001. H/R, hypoxia–reoxygenation; I/R, ischemia–reperfusion; EX, exercise preconditioning; KO, knockout; NC, matched negative control; SH, knockdown; OE, overexpression.

**Table S1. Baseline characteristics of the participants**

| **Groups** | **Control (*n* = 17)** | **Exercise (*n* = 17)** | ***t*, *df*** | ***P*-value** |
| --- | --- | --- | --- | --- |
| **Age, years** | 44 (8) | 45 (10) | *t* = 0.0935 *df* = 32 | 0.9261 |
| **Height, cm** | 172.00 (5.37) | 173.12 (4.55) | *t* = 0.6542 *df* = 32 | 0.5176 |
| **Weight, kg** | 78.35 (12.66) | 71.82 (9.79) | *t* = 1.6821 *df* = 32 | 0.1023 |
| **BMI, kg/m²** | 26.61 (4.92) | 23.92 (2.69) | *t* = 1.9761 *df* = 32 | 0.0568 |
| **VO₂max, mL/min/kg** | 26.2 (2.6) | 45.3 (5.9) | *t* = 12.1600 *df* = 32 | <0.0001 |
| **RERmax** | 1.27 (0.11) | 1.18 (0.11) | *t* = 1.8990 *df* = 32 | 0.0666 |
| **AT, mL/min/kg** | 12.2 (1.74) | 28.9 (5.1) | *t* = 12.7900 *df* = 32 | <0.0001 |

Data are presented as mean (SD). BMI was calculated for each participant as body weight (kg) divided by height squared (m²). Between-group comparisons used two-sided independent-samples Student's t-tests. BMI, body mass index; df, degrees of freedom; VO₂max, maximal oxygen consumption; RERmax, maximal respiratory exchange ratio; AT, anaerobic threshold.

Table S2. Results of two-way ANOVA

Figs. 1 and S1: I/R status × exercise; Figs. 3 and S2a–b: Igfbp2 knockout × exercise; Figs. S2e–i and S3e–h: IGFBP2 treatment × Gsdme genotype. Ordinary two-way ANOVA, n=8 per cell.

| **Figure / outcome** | **Effect** | **df1** | **df2** | **F value** | **P value** | **Partial η²** |
| --- | --- | --- | --- | --- | --- | --- |
| **Fig. S1c / Cleaved caspase 3** | I/R status | 1 | 28 | 293.575 | 2.245E-16 | 0.913 |
| *Same as above* | Exercise | 1 | 28 | 22.350 | 5.840E-05 | 0.444 |
| *Same as above* | I/R status × exercise | 1 | 28 | 39.588 | 8.374E-07 | 0.586 |
| **Fig. 1b / EF** | I/R status | 1 | 28 | 5192.233 | 2.444E-33 | 0.995 |
| *Same as above* | Exercise | 1 | 28 | 147.059 | 1.162E-12 | 0.840 |
| *Same as above* | I/R status × exercise | 1 | 28 | 72.536 | 2.935E-09 | 0.721 |
| **Fig. 1b / FS** | I/R status | 1 | 28 | 146.887 | 1.178E-12 | 0.840 |
| *Same as above* | Exercise | 1 | 28 | 18.685 | 0.0002 | 0.400 |
| *Same as above* | I/R status × exercise | 1 | 28 | 8.076 | 0.0083 | 0.224 |
| **Fig. S1c / GSDME** | I/R status | 1 | 28 | 213.174 | 1.279E-14 | 0.884 |
| *Same as above* | Exercise | 1 | 28 | 15.799 | 0.0004 | 0.361 |
| *Same as above* | I/R status × exercise | 1 | 28 | 29.901 | 7.725E-06 | 0.516 |
| **Fig. 1b / LVIDd** | I/R status | 1 | 28 | 56.598 | 3.404E-08 | 0.669 |
| *Same as above* | Exercise | 1 | 28 | 12.772 | 0.0013 | 0.313 |
| *Same as above* | I/R status × exercise | 1 | 28 | 6.205 | 0.0189 | 0.181 |
| **Fig. 1b / LVIDs** | I/R status | 1 | 28 | 462.408 | 6.009E-19 | 0.943 |
| *Same as above* | Exercise | 1 | 28 | 17.076 | 0.0003 | 0.379 |
| *Same as above* | I/R status × exercise | 1 | 28 | 21.565 | 7.342E-05 | 0.435 |
| **Fig. S1a / Masson** | I/R status | 1 | 28 | 4629.430 | 1.207E-32 | 0.994 |
| *Same as above* | Exercise | 1 | 28 | 348.054 | 2.494E-17 | 0.926 |
| *Same as above* | I/R status × exercise | 1 | 28 | 282.078 | 3.743E-16 | 0.910 |

| **Figure / outcome** | **Effect** | **df1** | **df2** | **F value** | **P value** | **Partial η²** |
| --- | --- | --- | --- | --- | --- | --- |
| **Fig. 1c / VO2** | I/R status | 1 | 28 | 499.805 | 2.143E-19 | 0.947 |
| *Same as above* | Exercise | 1 | 28 | 52.766 | 6.585E-08 | 0.653 |
| *Same as above* | I/R status × exercise | 1 | 28 | 12.601 | 0.0014 | 0.310 |
| **Fig. 1d / TTC** | I/R status | 1 | 28 | 1881.121 | 3.206E-27 | 0.985 |
| *Same as above* | Exercise | 1 | 28 | 319.681 | 7.501E-17 | 0.919 |
| *Same as above* | I/R status × exercise | 1 | 28 | 353.487 | 2.039E-17 | 0.927 |
| **Fig. S1b / TUNEL** | I/R status | 1 | 28 | 267.062 | 7.517E-16 | 0.905 |
| *Same as above* | Exercise | 1 | 28 | 32.033 | 4.590E-06 | 0.534 |
| *Same as above* | I/R status × exercise | 1 | 28 | 20.526 | 9.996E-05 | 0.423 |
| **Fig. 1h / Individual mitochondrial area** | I/R status | 1 | 28 | 780.801 | 5.396E-22 | 0.965 |
| *Same as above* | Exercise | 1 | 28 | 30.516 | 6.633E-06 | 0.522 |
| *Same as above* | I/R status × exercise | 1 | 28 | 3.548 | 0.0700 | 0.112 |
| **Fig. 1h / Average mitochondrial number** | I/R status | 1 | 28 | 60.202 | 1.881E-08 | 0.683 |
| *Same as above* | Exercise | 1 | 28 | 13.147 | 0.0011 | 0.320 |
| *Same as above* | I/R status × exercise | 1 | 28 | 11.217 | 0.0023 | 0.286 |
| **Fig. 3f / AKT** | Igfbp2 KO | 1 | 28 | 0.965 | 0.3345 | 0.033 |
| *Same as above* | Exercise | 1 | 28 | 0.936 | 0.3416 | 0.032 |
| *Same as above* | Igfbp2 KO × exercise | 1 | 28 | 1.111 | 0.3009 | 0.038 |
| **Fig. 3f / CAS3** | Igfbp2 KO | 1 | 28 | 36.872 | 1.507E-06 | 0.568 |
| *Same as above* | Exercise | 1 | 28 | 31.801 | 4.852E-06 | 0.532 |
| *Same as above* | Igfbp2 KO × exercise | 1 | 28 | 27.863 | 1.295E-05 | 0.499 |

| **Figure / outcome** | **Effect** | **df1** | **df2** | **F value** | **P value** | **Partial η²** |
| --- | --- | --- | --- | --- | --- | --- |
| **Fig. 3a / EF** | Igfbp2 KO | 1 | 28 | 21.183 | 8.217E-05 | 0.431 |
| *Same as above* | Exercise | 1 | 28 | 32.810 | 3.814E-06 | 0.540 |
| *Same as above* | Igfbp2 KO × exercise | 1 | 28 | 27.192 | 1.543E-05 | 0.493 |
| **Fig. 3a / FS** | Igfbp2 KO | 1 | 28 | 103.969 | 6.253E-11 | 0.788 |
| *Same as above* | Exercise | 1 | 28 | 124.506 | 8.120E-12 | 0.816 |
| *Same as above* | Igfbp2 KO × exercise | 1 | 28 | 101.493 | 8.171E-11 | 0.784 |
| **Fig. 3f / GSDME** | Igfbp2 KO | 1 | 28 | 41.776 | 5.309E-07 | 0.599 |
| *Same as above* | Exercise | 1 | 28 | 92.404 | 2.285E-10 | 0.767 |
| *Same as above* | Igfbp2 KO × exercise | 1 | 28 | 61.707 | 1.479E-08 | 0.688 |
| **Fig. 3a / LVIDd** | Igfbp2 KO | 1 | 28 | 4.736 | 0.0381 | 0.145 |
| *Same as above* | Exercise | 1 | 28 | 2.266 | 0.1434 | 0.075 |
| *Same as above* | Igfbp2 KO × exercise | 1 | 28 | 13.011 | 0.0012 | 0.317 |
| **Fig. 3a / LVIDs** | Igfbp2 KO | 1 | 28 | 1.829 | 0.1870 | 0.061 |
| *Same as above* | Exercise | 1 | 28 | 0.192 | 0.6645 | 0.007 |
| *Same as above* | Igfbp2 KO × exercise | 1 | 28 | 13.702 | 0.0009 | 0.329 |
| **Fig. S2a / Masson** | Igfbp2 KO | 1 | 28 | 33.896 | 2.958E-06 | 0.548 |
| *Same as above* | Exercise | 1 | 28 | 33.990 | 2.894E-06 | 0.548 |
| *Same as above* | Igfbp2 KO × exercise | 1 | 28 | 33.662 | 3.123E-06 | 0.546 |

| **Figure / outcome** | **Effect** | **df1** | **df2** | **F value** | **P value** | **Partial η²** |
| --- | --- | --- | --- | --- | --- | --- |
| **Fig. 3f / p-AKT** | Igfbp2 KO | 1 | 28 | 53.550 | 5.738E-08 | 0.657 |
| *Same as above* | Exercise | 1 | 28 | 95.658 | 1.567E-10 | 0.774 |
| *Same as above* | Igfbp2 KO × exercise | 1 | 28 | 63.196 | 1.171E-08 | 0.693 |
| **Fig. 3f / p-PI3K** | Igfbp2 KO | 1 | 28 | 62.406 | 1.325E-08 | 0.690 |
| *Same as above* | Exercise | 1 | 28 | 80.847 | 9.528E-10 | 0.743 |
| *Same as above* | Igfbp2 KO × exercise | 1 | 28 | 41.883 | 5.193E-07 | 0.599 |
| **Fig. 3f / PDCD4** | Igfbp2 KO | 1 | 28 | 81.444 | 8.817E-10 | 0.744 |
| *Same as above* | Exercise | 1 | 28 | 57.824 | 2.774E-08 | 0.674 |
| *Same as above* | Igfbp2 KO × exercise | 1 | 28 | 86.534 | 4.633E-10 | 0.756 |
| **Fig. 3b / VO2** | Igfbp2 KO | 1 | 28 | 32.415 | 4.189E-06 | 0.537 |
| *Same as above* | Exercise | 1 | 28 | 17.949 | 0.0002 | 0.391 |
| *Same as above* | Igfbp2 KO × exercise | 1 | 28 | 16.628 | 0.0003 | 0.373 |
| **Fig. 3f / PI3K** | Igfbp2 KO | 1 | 28 | 0.006 | 0.9403 | 0.000 |
| *Same as above* | Exercise | 1 | 28 | 1.428 | 0.2421 | 0.049 |
| *Same as above* | Igfbp2 KO × exercise | 1 | 28 | 2.871 | 0.1013 | 0.093 |
| **Fig. 3c / TTC** | Igfbp2 KO | 1 | 28 | 179.974 | 1.027E-13 | 0.865 |
| *Same as above* | Exercise | 1 | 28 | 167.542 | 2.446E-13 | 0.857 |
| *Same as above* | Igfbp2 KO × exercise | 1 | 28 | 190.067 | 5.274E-14 | 0.872 |

| **Figure / outcome** | **Effect** | **df1** | **df2** | **F value** | **P value** | **Partial η²** |
| --- | --- | --- | --- | --- | --- | --- |
| **Fig. S2b / TUNEL** | Igfbp2 KO | 1 | 28 | 47.986 | 1.571E-07 | 0.632 |
| *Same as above* | Exercise | 1 | 28 | 104.007 | 6.227E-11 | 0.788 |
| *Same as above* | Igfbp2 KO × exercise | 1 | 28 | 145.719 | 1.295E-12 | 0.839 |
| **Fig. 3g / Cardiac IGFBP2 WB** | Igfbp2 KO | 1 | 28 | 1369.748 | 2.528E-25 | 0.980 |
| *Same as above* | Exercise | 1 | 28 | 108.840 | 3.748E-11 | 0.795 |
| *Same as above* | Igfbp2 KO × exercise | 1 | 28 | 120.862 | 1.142E-11 | 0.812 |
| **Fig. 3h / Individual mitochondrial area** | Igfbp2 KO | 1 | 28 | 30.096 | 7.359E-06 | 0.518 |
| *Same as above* | Exercise | 1 | 28 | 35.852 | 1.892E-06 | 0.561 |
| *Same as above* | Igfbp2 KO × exercise | 1 | 28 | 20.820 | 9.153E-05 | 0.426 |
| **Fig. 3h / Average mitochondrial number** | Igfbp2 KO | 1 | 28 | 24.947 | 2.814E-05 | 0.471 |
| *Same as above* | Exercise | 1 | 28 | 17.766 | 0.0002 | 0.388 |
| *Same as above* | Igfbp2 KO × exercise | 1 | 28 | 18.318 | 0.0002 | 0.395 |
| **Fig. S2h / AKT** | IGFBP2 treatment | 1 | 28 | 0.069 | 0.7945 | 0.002 |
| *Same as above* | Gsdme genotype | 1 | 28 | 1.106 | 0.3019 | 0.038 |
| *Same as above* | IGFBP2 treatment × Gsdme genotype | 1 | 28 | 0.069 | 0.7945 | 0.002 |

| **Figure / outcome** | **Effect** | **df1** | **df2** | **F value** | **P value** | **Partial η²** |
| --- | --- | --- | --- | --- | --- | --- |
| **Fig. S2e / CCK8** | IGFBP2 treatment | 1 | 28 | 110.337 | 3.214E-11 | 0.798 |
| *Same as above* | Gsdme genotype | 1 | 28 | 154.693 | 6.370E-13 | 0.847 |
| *Same as above* | IGFBP2 treatment × Gsdme genotype | 1 | 28 | 128.566 | 5.605E-12 | 0.821 |
| **Fig. S2i / Cleaved-caspase 3** | IGFBP2 treatment | 1 | 28 | 39.649 | 8.266E-07 | 0.586 |
| *Same as above* | Gsdme genotype | 1 | 28 | 60.279 | 1.858E-08 | 0.683 |
| *Same as above* | IGFBP2 treatment × Gsdme genotype | 1 | 28 | 52.924 | 6.404E-08 | 0.654 |
| **Fig. S3h / JC-1-a** | IGFBP2 treatment | 1 | 28 | 15.578 | 0.0005 | 0.357 |
| *Same as above* | Gsdme genotype | 1 | 28 | 16.761 | 0.0003 | 0.374 |
| *Same as above* | IGFBP2 treatment × Gsdme genotype | 1 | 28 | 12.922 | 0.0012 | 0.316 |
| **Fig. S3h / JC-1-m** | IGFBP2 treatment | 1 | 28 | 11.228 | 0.0023 | 0.286 |
| *Same as above* | Gsdme genotype | 1 | 28 | 11.759 | 0.0019 | 0.296 |
| *Same as above* | IGFBP2 treatment × Gsdme genotype | 1 | 28 | 11.217 | 0.0023 | 0.286 |
| **Fig. S2i / N-GSDME** | IGFBP2 treatment | 1 | 28 | 475.603 | 4.140E-19 | 0.944 |
| *Same as above* | Gsdme genotype | 1 | 28 | 532.175 | 9.301E-20 | 0.950 |
| *Same as above* | IGFBP2 treatment × Gsdme genotype | 1 | 28 | 514.869 | 1.444E-19 | 0.948 |
| **Fig. S3e / OXPHOS** | IGFBP2 treatment | 1 | 28 | 10.256 | 0.0034 | 0.268 |
| *Same as above* | Gsdme genotype | 1 | 28 | 27.537 | 1.410E-05 | 0.496 |
| *Same as above* | IGFBP2 treatment × Gsdme genotype | 1 | 28 | 15.120 | 0.0006 | 0.351 |

| **Figure / outcome** | **Effect** | **df1** | **df2** | **F value** | **P value** | **Partial η²** |
| --- | --- | --- | --- | --- | --- | --- |
| **Fig. S2i / p-AKT** | IGFBP2 treatment | 1 | 28 | 166.750 | 2.590E-13 | 0.856 |
| *Same as above* | Gsdme genotype | 1 | 28 | 155.496 | 5.988E-13 | 0.847 |
| *Same as above* | IGFBP2 treatment × Gsdme genotype | 1 | 28 | 147.313 | 1.139E-12 | 0.840 |
| **Fig. S2i / p-PI3K** | IGFBP2 treatment | 1 | 28 | 281.457 | 3.850E-16 | 0.910 |
| *Same as above* | Gsdme genotype | 1 | 28 | 218.034 | 9.664E-15 | 0.886 |
| *Same as above* | IGFBP2 treatment × Gsdme genotype | 1 | 28 | 196.336 | 3.540E-14 | 0.875 |
| **Fig. S2i / PDCD4** | IGFBP2 treatment | 1 | 28 | 244.185 | 2.336E-15 | 0.897 |
| *Same as above* | Gsdme genotype | 1 | 28 | 308.251 | 1.199E-16 | 0.917 |
| *Same as above* | IGFBP2 treatment × Gsdme genotype | 1 | 28 | 291.536 | 2.454E-16 | 0.912 |
| **Fig. S2h / PI3K** | IGFBP2 treatment | 1 | 28 | 0.009 | 0.9238 | 0.000 |
| *Same as above* | Gsdme genotype | 1 | 28 | 1.575 | 0.2198 | 0.053 |
| *Same as above* | IGFBP2 treatment × Gsdme genotype | 1 | 28 | 0.233 | 0.6330 | 0.008 |
| **Fig. S3f / ROS** | IGFBP2 treatment | 1 | 28 | 28.282 | 1.163E-05 | 0.503 |
| *Same as above* | Gsdme genotype | 1 | 28 | 45.216 | 2.669E-07 | 0.618 |
| *Same as above* | IGFBP2 treatment × Gsdme genotype | 1 | 28 | 26.614 | 1.796E-05 | 0.487 |
| **Fig. S2g / TUNEL** | IGFBP2 treatment | 1 | 28 | 342.259 | 3.101E-17 | 0.924 |
| *Same as above* | Gsdme genotype | 1 | 28 | 253.888 | 1.428E-15 | 0.901 |
| *Same as above* | IGFBP2 treatment × Gsdme genotype | 1 | 28 | 291.199 | 2.491E-16 | 0.912 |

**Note.** df1, numerator degrees of freedom; df2, denominator degrees of freedom. P values are unadjusted omnibus-test values. The second column level in the Fig. 3h individual mitochondrial area source file is labeled ‘individual’ rather than ‘EX’; this label does not affect the calculation and should be confirmed before final figure preparation.

Supplementary Methods

This supplementary document summarizes the sample-processing, data-independent acquisition mass-spectrometry, database-search, quantitative normalization, false-discovery-rate adjustment, expression-pattern clustering, and functional-enrichment procedures used for the human-sample proteomic data set (project XA01170DA) and the mouse-heart proteomic data set (project XC00584DA).

1. Human-sample proteomic workflow

1.1 Sample preparation and depletion of high-abundance proteins

Human plasma samples were retrieved from storage at −80 °C and centrifuged at 12,000 × g for 10 min at 4 °C to remove cellular debris. The supernatant was transferred to a new tube, and high-abundance proteins were depleted using Pierce Top 14 Abundant Protein Depletion Spin Columns (Thermo Fisher Scientific; Cat. No. A36370) according to the manufacturer’s instructions. Protein concentration was determined using a BCA protein assay kit (Beyotime; Cat. No. P0011).

1.2 Filter-aided protein digestion and peptide desalting

Equal amounts of protein from each sample were adjusted to the same volume with lysis buffer. Proteins were reduced with 5 mM dithiothreitol (Sigma-Aldrich; Cat. No. D9163-25G) for 30 min at 56 °C and alkylated with 11 mM iodoacetamide (Sigma-Aldrich; Cat. No. V900335-5G) for 15 min at room temperature in the dark. The alkylated samples were transferred to 0.5-mL, 10-kDa ultrafiltration devices (Millipore; Cat. No. UFC501024). Samples were exchanged three times with 8 M urea (Sigma-Aldrich; Cat. No. V900119-500G) and then three times with 200 mM triethylammonium bicarbonate, with centrifugation at 12,000 × g for 20 min at room temperature. Trypsin (Promega; Cat. No. V5117) was added at a trypsin-to-protein mass ratio of 1:50, and digestion proceeded overnight. Peptides were recovered by centrifugation at 12,000 × g for 10 min at room temperature, followed by a second recovery with ddH₂O. The two peptide fractions were pooled.

The digested peptides were acidified to pH 2–3 with 10% trifluoroacetic acid (Sigma-Aldrich; Cat. No. 302031-1L) and centrifuged at 12,000 × g for 10 min at room temperature. The supernatant was transferred to a new tube for StageTip desalting using Pierce C18 Tips with a 100-µL bed volume (Thermo Scientific; Cat. No. 87784). The StageTip was activated with 50 µL activation solution and centrifuged at 1,500 × g for 1 min, followed by two equilibration steps with 50 µL desalting solution at 1,500 × g for 1 min. A 50-µL peptide sample was loaded and centrifuged at 1,500 × g for 1 min. The StageTip was washed twice with 50 µL desalting solution at 1,500 × g for 1 min, transferred to a new tube, and eluted twice with 20 µL elution solution at 750 × g for 1 min. The two eluates were pooled and vacuum-dried for subsequent analysis.

1.3 Liquid chromatography–tandem mass spectrometry

Tryptic peptides were dissolved in mobile phase A and loaded directly onto a home-made reversed-phase analytical column (25 cm length, 100 µm inner diameter) coupled to a Vanquish neo UHPLC system (Thermo Fisher Scientific, Waltham, MA, USA). Mobile phase A consisted of 0.1% formic acid and 2% acetonitrile in water, whereas mobile phase B consisted of 0.1% formic acid and 90% acetonitrile in water. Peptides were separated at 500 nL/min using the following gradient: 7%–20% B from 0 to 16 min, 20%–32% B from 16 to 24 min, 32%–80% B from 24 to 27 min, and 80% B from 27 to 30 min.

The separated peptides were ionized using a nano-electrospray ion source and analyzed on an Orbitrap Exploris 480 mass spectrometer (Thermo Fisher Scientific, Waltham, MA, USA). The spray voltage was 2,300 V, and the FAIMS compensation voltages were −45 and −70 V. Data were acquired in data-independent acquisition mode. Full MS scans were acquired over m/z 390–810 at a resolution of 30,000. For MS/MS scans, the fixed first mass was m/z 200 and the resolution was 30,000. Higher-energy collisional dissociation was performed using normalized collision energies of 25%, 30%, and 35%. The automatic gain-control target was 3 × 10⁶, and the maximum injection time was set to Auto.

1.4 Database search and identification control

DIA data were processed using Spectronaut version 17 (Biognosys) with the embedded Pulsar search engine and the software default parameters. Tandem mass spectra were searched against the Homo_sapiens_9606_SP_20230103.fasta database containing 20,389 sequences, concatenated with a reverse decoy database. Trypsin/P was specified as the cleavage enzyme, allowing up to two missed cleavages. Carbamidomethylation of cysteine was specified as a fixed modification, whereas methionine oxidation and protein N-terminal acetylation were specified as variable modifications. False discovery rates at the protein, peptide, and peptide-spectrum-match levels were controlled at 1%.

2. Mouse-heart proteomic workflow

2.1 Protein extraction

Mouse-heart samples were removed from storage at −80 °C and ground to a fine powder in a liquid-nitrogen-precooled mortar. The powder was transferred to a 5-mL centrifuge tube and mixed with four volumes of lysis buffer containing 1% Triton X-100, 1% Protease Inhibitor Cocktail III (Merck Millipore; Cat. No. 539134-10ML), 1% phosphatase inhibitor (Selleck.cn; Cat. No. B15002), 3 µM trichostatin A (MedChemExpress; Cat. No. HY-15144), and 50 mM nicotinamide (Sigma-Aldrich; Cat. No. N0636-500G). Samples were sonicated on ice for 3 min using a high-intensity ultrasonic processor and centrifuged at 12,000 × g for 10 min at 4 °C. The supernatant was transferred to a fresh tube, and protein concentration was determined using a BCA assay kit (Beyotime; Cat. No. P0011).

2.2 Protein precipitation, digestion, reduction, alkylation, and peptide desalting

Equal amounts of protein were used for each sample, and sample volumes were adjusted with lysis buffer. Trichloroacetic acid (Sigma-Aldrich; Cat. No. T4885-2KG) was slowly added to a final concentration of 20% (m/v). Samples were vortexed and incubated for 2 h at 4 °C. Protein precipitates were collected by centrifugation at 4,500 × g for 5 min at 4 °C, washed three times with prechilled acetone, and dried for 1 min. The precipitates were redissolved in 200 mM triethylammonium bicarbonate (Sigma-Aldrich; Cat. No. T7408-500ML) and dispersed by sonication. Trypsin (Promega; Cat. No. V5117) was added at a trypsin-to-protein mass ratio of 1:50, and digestion was performed overnight. Samples were subsequently reduced with 5 mM dithiothreitol (Sigma-Aldrich; Cat. No. D9163-25G) for 60 min at 37 °C and alkylated with 11 mM iodoacetamide (Sigma-Aldrich; Cat. No. V900335-5G) for 45 min at room temperature in the dark. The resulting peptides were desalted using a Strata X solid-phase extraction column.

2.3 dia-PASEF LC–MS/MS analysis

Tryptic peptides were dissolved in mobile phase A and loaded directly onto a home-made reversed-phase analytical column (25 cm length, 100 µm inner diameter). Mobile phase A consisted of 0.1% formic acid and 2% acetonitrile in water, and mobile phase B consisted of 0.1% formic acid in acetonitrile. Peptides were separated using a nanoElute UHPLC system (Bruker, Karlsruhe, Germany) at 500 nL/min with the following gradient: 6%–24% B from 0 to 14 min, 24%–35% B from 14 to 16 min, 35%–80% B from 16 to 18 min, and 80% B from 18 to 20 min.

Eluted peptides were ionized through a capillary source and analyzed using a timsTOF Pro 2 mass spectrometer (Bruker, Karlsruhe, Germany). The electrospray voltage was 1.75 kV, and precursor and fragment ions were detected by time-of-flight analysis. Data were acquired in data-independent parallel accumulation–serial fragmentation (dia-PASEF) mode. The full MS scan range was m/z 300–1,500. Twenty PASEF MS/MS scans were acquired per cycle. The MS/MS scan range was m/z 400–850, with a 7 m/z isolation window.

2.4 Database search and identification control

DIA data were processed using DIA-NN version 1.8 with the software default parameters unless otherwise specified. Spectra were searched against the Mus_musculus_10090_SP_20241202.fasta database containing 17,236 sequences. Trypsin/P was specified as the cleavage enzyme, with up to one missed cleavage allowed. N-terminal methionine excision and cysteine carbamidomethylation were specified as fixed modifications. A theoretical spectral library was generated using the DIA-NN deep-learning algorithm. A reverse decoy database was included to estimate false-positive identifications, and the precursor-level false discovery rate was controlled at 1%.

3. Quantitative normalization and multiple-testing adjustment

For both proteomic projects, the raw LC–MS data were converted into protein-intensity matrices. Normalized intensity represented raw intensity after correction of sample and batch effects. The normalized intensity for sample i and protein j (Iij) was transformed to a relative quantitative value (Rij) by centering each protein to its mean intensity across the samples included in the corresponding matrix:

R_ij_ = I_ij_ / Mean(I_j_)

Original P values were adjusted separately within each pairwise comparison using the Benjamini–Hochberg procedure to provide false-discovery-rate values. Adjusted values were reported in the FDR columns of the supplied workbooks. The human data workbook contains FDR values for T1/C, T2/C, T3/C, T2/T1, T3/T1, and T3/T2; the mouse-heart workbook contains FDR values for EL/CL, ER/CR, CR/CL, and ER/EL. Comparisons without an available P value were recorded as NA.

4. Temporal expression-pattern clustering

Expression-pattern clustering was performed for the human temporal proteomic data set. Relative protein-abundance values were log₂-transformed, and proteins with a standard deviation greater than 0.2 across the C, T1, T2, and T3 sample groups were retained. Of the 1,704 identified proteins, 924 passed this variability filter and were included in clustering. The Mfuzz implementation of fuzzy c-means clustering was applied with a prespecified cluster number of k = 7 and a fuzzification coefficient of m = 2, producing seven temporal expression-pattern clusters. Each protein received a membership value for each cluster, allowing overlapping rather than strictly binary cluster assignment. Cluster results were displayed as relative-abundance line plots and heat maps across the four sample groups.

5. Functional-enrichment analysis

Proteins assigned to each expression-pattern cluster were analyzed separately for Kyoto Encyclopedia of Genes and Genomes pathways, Gene Ontology Biological Process, Gene Ontology Cellular Component, Gene Ontology Molecular Function, and protein-domain enrichment. Enrichment significance was assessed using Fisher’s exact test, with a nominal P value < 0.05 considered significant in the supplied report. Fold enrichment was calculated as (Mapping/All.mapping)/(Background/All.background), where Mapping denotes the number of proteins from a specified cluster annotated to a term, All.mapping denotes the total number of annotated proteins in that cluster, Background denotes the number of proteins from all clusters annotated to the term, and All.background denotes the total number of annotated proteins across all clusters. For the summary display, the two most significantly enriched entries from each enrichment output were shown beside the corresponding expression-pattern cluster.

6. Proteomic identification and quantification summary

Table S3 summarizes peptide and protein identification and the proteins available for quantitative comparison in the human plasma and mouse-heart proteomic datasets, providing an overview of proteomic coverage. Table S4 documents the acquisition platforms, database-search settings, and identification-level false discovery rate criteria to support methodological transparency and reproducibility.

Table S3. Identification and quantification summary for the two proteomic data sets.

| Data set | Identified peptides | Unique peptides | Identified proteins | Comparable proteins |
| --- | --- | --- | --- | --- |
| Human samples (XA01170DA) | 11,348 | 10,540 | 1,704 | 1,640 |
| Mouse heart (XC00584DA) | 38,772 | 38,189 | 5,371 | 5,342 |

Table S4. Principal acquisition and database-search settings.

| Data set | LC–MS platform | Search software and database | Identification FDR |
| --- | --- | --- | --- |
| Human samples | Vanquish neo UHPLC; Orbitrap Exploris 480; DIA | Spectronaut v17/Pulsar; Homo_sapiens_9606_SP_20230103.fasta (20,389 sequences) | Protein, peptide, and PSM levels controlled at 1% |
| Mouse heart | nanoElute UHPLC; timsTOF Pro 2; dia-PASEF | DIA-NN v1.8; Mus_musculus_10090_SP_20241202.fasta (17,236 sequences) | Precursor level controlled at 1% |
